# Selenium-Enriched Rapeseed Extract Synergizes with Chemotherapy Drug Cisplatin in Inhibiting Proliferation and Promoting Apoptosis of Colorectal Cancer Cells

**DOI:** 10.3390/cells15211934

**Published:** 2026-10-24

**Authors:** Xiaofei Duan, Yiqing Lu, Hongyu Zhou, Zi Zhang, Zhanan Zhou, Miao Wang, Zhenna Chen, Xiaoling Dun, Yang Zhu, Hanzhong Wang, Lixi Jiang

**Affiliations:** 1 Institute of Crop Science, Zhejiang University, Hangzhou 310058, China; 12316129@zju.edu.cn (X.D.);; 2 Key Laboratory of Biology and Genetic Improvement of Oil Crops, Ministry of Agriculture and Rural Affairs, Oil Crops Research Institute of the Chinese Academy of Agricultural Sciences, Wuhan 430062, China; 3 Hubei Hongshan Laboratory, Wuhan 430070, China

**Keywords:** Selenium-enriched rapeseed, CDDP, HPLC-ICP-MS, CRC, integrated multi-omics analysis

## Abstract

**Highlights:**

**What are the main findings?**

- Water extract (WE) of selenium-enriched rapeseed can significantly enhance the effect of cisplatin (CDDP) on colorectal cancer cells without exerting significant toxic side effects on normal cells such as NCM460 and HK-2.
- Methylselenocysteine (MSC), sulforaphane (SFN), celastrol (Cel), indole-3-carbinol (I3C), α-linolenic acid (ALA), and linoleic acid (LA) in the WE can act as synergistic active monomers.

**What are the implications of the main findings?**

- Multi-omics and molecular simulations help to uncover the targets and regulatory pathways underlying the synergistic effect of the WE and CDDP on inhibiting cancer cell growth.
- Selenium-enriched rapeseed and cisplatin (CDDP) exert synergistic antitumor activity, and they may provide a new dietary combination therapy to overcome drug resistance.

**Abstract:**

Chemotherapy treatment of colorectal cancer (CRC) with cisplatin (CDDP) faces drug resistance and cytotoxicity issues, highlighting the urgent need for combination strategies. Selenium-enriched rapeseed extracts exhibit anticancer effects; however, their bioactive components and mechanisms remain unclear. Here, we applied different solvents to fractionate extracts of selenium-enriched rapeseed and found that the water extract (WE) fraction significantly enhanced the cytotoxic effect of CDDP on cancer cells without damaging normal cells, namely, HK-2 and NCM460. High-performance liquid chromatography–inductively coupled plasma–mass spectrometry (HPLC-ICP-MS) analysis revealed methylselenocysteine (MSC) and selenocysteine (SeCys_2_) as the main selenium species in the WE. Through cell biology and integrative multi-omics analysis, we found a synergistic anti-CRC cell effect upon combining CDDP with MSC, sulforaphane (SFN), celastrol (Cel), indole-3-carbinol (I3C), α-linolenic acid (ALA) or linoleic acid (LA). We propose that CDDP-WE (cisplatin and water extract) combination treatment holds promise for improving antitumor efficacy against chemo-refractory cells.

## 1. Introduction

Colorectal cancer (CRC) is one of the most common cancers in many countries [1]. It is also a leading cause of death, ranking second only to lung cancer [2]. Various methods are available for treating CRC, including traditional surgery, radiotherapy, chemotherapy, targeted therapy, and immunotherapy [3,4,5]. However, the cytotoxicity of chemotherapy drugs to normal cells limits their use in clinical application [6]. Therefore, there is a need to develop natural products that can precisely target colorectal cells and produce synergistic effects with chemotherapy drugs to enhance antitumor effects, and that have low cytotoxicity, as they have good prospects for practical application [7].

Since the discovery of the activity of platinum-based drugs, one of the first generations of such drugs, cisplatin (CDDP), has been used in cancer treatment, usually in conjunction with other therapies, such as surgery [8], radiation [9] or immunotherapy [10]. Although CDDP can be effective in treating CRC, its clinical application is limited, particularly because of its nephrotoxicity [11]. Natural product extracts have long been a unique source of chemotherapeutics used in the combination treatment of cancer [12]. Combination strategies are essential not only for enhancing synergistic anticancer effects but also for alleviating cytotoxicity in healthy tissues through dose reduction [13].

Many diet-based natural products have emerged as complementary and potential sources of novel anticancer adjuvant drugs because of their overall safety and synergism with conventional chemotherapy in the treatment of various cancers. Selenium-enriched rapeseed is a vegetable with medicinal value, and it is rich in various active ingredients, such as selenium, polyphenols [14], flavonoids [15], and fatty acids [16], which endow it with diverse biological activities. The glucosinolates in sprouts can produce isothiocyanates with anticancer effects in vivo [17]. In addition, selenium-enriched rapeseed exerts certain protective effects, as it can improve male fertility [18] and alleviate cadmium-induced reproductive toxicity [19]. Previous studies have shown that selenium-enriched rapeseed extracts exert anticancer activity against triple-negative breast cancer by triggering resolvin D5 (RvD5) activation and restraining IL-17 signaling [20]. In addition, rapeseed is rich in alpha-linolenic acid, a type of bioactive fatty acid with documented anticancer properties. The combined administration of eicosapentaenoic acid (EPA) and docosahexaenoic acid (DHA) can alleviate the nephrotoxicity caused by CDDP [21]. The α-linolenic acid (ALA) can suppress tumor cell metastasis and trigger apoptotic cell death in oral squamous cell carcinoma [16]; it also inhibits the proliferation of hepatocellular carcinoma cells by modulating the Farnesoid X receptor/β-catenin signaling axis [22]. Collectively, these findings highlight that both selenium compounds and endogenous fatty acids in selenium-enriched rapeseed possess promising antitumor bioactivity. Selenium-enriched rapeseed has attracted considerable attention because of its high selenium content. Selenocysteine (SeCys_2_), methylselenocysteine (MSC), selenomethionine (SeMet), selenite (SeIV), and selenate (SeVI) are considered to be the main forms in rapeseed, as revealed by Zhan et al. [23]. However, the active anticancer compounds of selenium-enriched rapeseed and their possible underlying impacts on and mechanisms in CRC cells remain largely unclear.

To identify the active anticancer compounds of selenium-enriched rapeseed and uncover their anticancer mechanisms when used either as monomers or in combination therapy in CRC treatment, we selected two common human colorectal cancer cell lines, namely, SW620 and HCT116 cells, as models that have been widely utilized in studies of CRC [24]. First, we optimized the extraction parameters, including extraction solvent, solvent concentration, solid–liquid ratio, and extraction time. Second, we identified the most effective fraction that exerted a synergistic anticancer effect in the CRC cell model. Third, we quantitatively analyzed the selenium species and contents in different fractions using high-performance liquid chromatography–inductively coupled plasma–mass spectrometry (HPLC-ICP-MS). Finally, we examined several monomers from selenium-enriched rapeseed for their independent anticancer efficacy and cytotoxicity, as well as the combinatorial effects of these monomers and CDDP against CRC cells. We found that MSC, sulforaphane (SFN), celastrol (Cel), Indole-3-carbinol (I3C), ALA, and linoleic acid (LA) exerted synergistic effects with CDDP against CRC cells in vitro. Our results suggest that combining CDDP with selenium-enriched rapeseed extract or derived monomers may provide an effective therapeutic approach for treating CRC by inhibiting cancer cell growth.

## 2. Materials and Methods

### 2.1. Chemicals and Reagents

Ethanol, petroleum ether, ethyl acetate, and n-butanol were purchased from China National Pharmaceutical Group Chemical Reagent Co., Ltd. (Beijing, China). Sodium selenite (Na_2_SeO_3_), sodium selenate (Na_2_SeO_4_), SeMet, SeCys_2_, and MSC were purchased from the China National Institute of Standard Material (Beijing, China). A Cell Counting Kit-8 (CCK-8) assay kit was obtained from Biosharp Co., Ltd. (Beijing, China). An Annexin V-FITC/PI Apoptosis Detection Kit and a cell cycle detection kit were purchased from Elabscience Biotechnology Co., Ltd. (Wuhan, China). Cisplatin, 2,2’-azino-bis-3ethylbenzthiazoline-6-sulphonate (ABTS), reactive oxygen species (ROS), L-lactate dehydrogenase (L-LDH), ALA, LA, Cel, SFN and I3C were provided by Beijing Solarbio Science and Technology Co., Ltd. (Beijing, China). Trypsin–EDTA (0.05%) was obtained from Genview (FL, USA). A mitochondrial membrane potential assay kit, a JC-1,5-ethynyl-2ʹ-deoxyuridine (EdU) cell proliferation kit, and radioimmunoprecipitation assay (RIPA) lysis buffer were obtained from Beyotime Biotechnology (Nanjing, China). A reduced glutathione (GSH) assay kit (microplate method) was purchased from Nanjing Jiancheng Bioengineering Institute (Nanjing, China). Primary antibodies against BAX, Bcl-2, Caspase-3, Caspase-9, p53, and β-actin were purchased from Proteintech (Wuhan, China). HRP-conjugated goat anti-rabbit IgG (H+L) secondary antibody (Cat No.SA00001-2, Proteintech, Wuhan, China) was applied as the secondary antibody.

### 2.2. Plant Materials

Selenium-enriched rapeseed flowering stalks were collected from the Oil Crops Research Institute, Chinese Academy of Agricultural Sciences (Wuhan, China). The collected flowering stalks were ground into powder, freeze-dried with a lyophilizer (Labconco, Kansas City, MO, USA) and then stored at 4 °C.

### 2.3. Optimization of Extraction Conditions

Single-factor experiments were conducted to identify the optimal conditions of solvent concentration, solid–liquid ratio, and extraction time based on the efficacy of selenium-enriched rapeseed flowering stalks alone and in combination with cisplatin on SW620 cells.

### 2.4. Bioactivity-Guided Fractionation

After determining the optimal extraction conditions, crude extract (CE) was obtained. Then four fractions, namely, petroleum ether extract (PEE), ethyl acetate extract (EAE), n-butanol extract (BE), and water extract (WE), were obtained via liquid–liquid partitioning [25]. Subsequently, PEE and EAE were dissolved in DMSO while the other fractions were directly dissolved in culture medium. The most active fraction was identified using the CCK-8 assay and the ZIP synergy score.

### 2.5. Chromatography-Based Detection Analysis

#### 2.5.1. HPLC-ICP-MS Analysis

The total selenium content and different selenium species in various fractions were detected using an HPLC-ICP-MS system (Agilent 7900, Agilent Technologies, Santa Clara, CA, USA) as described [23]. First, 50 mg of freeze-dried extracts and 5 mL of nitric acid were added to DigiBlock (LabTech, Beijing, China) to determine total selenium content. The mixture was digested at 85 °C for 3 h, followed by evaporation at 120 °C to near dryness. It was then dissolved with 3 mL of deionized water and introduced into ICP-MS. For analysis of selenium species, 20 mg of extracts was incubated with 2 mL of protease XIV solution (10 mg/mL) and 18 mL of ultrapure water in individual tubes. After 40 min of ultrasonic extraction, samples were centrifuged at 12,000 rpm for 15 min. The resulting supernatant was subjected to HPLC–ICP-MS, enabling the separation and identification of five selenium species.

#### 2.5.2. Liquid Chromatography-Tandem Mass Spectrometry (LC-MS/MS) Analysis

Cell samples were obtained after 24 h of treatment with CDDP and WE alone or in combination. Tryptic peptides were dissolved in solvent A. The digested sample was loaded onto a homemade reversed-phase analytical column (25 cm length, 100 μm i.d.), following the protocol of the manufacturer. The peptides were subjected to capillary source followed by timsTOF Pro mass spectrometry. The timsTOF Pro was operated in data-independent parallel accumulation-serial fragmentation (dia-PASEF) mode. The full MS scan was set to 300–1500 (MS/MS scan range), and 20 PASEF (MS/MS mode)-MS/MS scans were obtained per cycle. The MS/MS scan range was set to 400–850, and the isolation window was set to 7 *m*/*z*.

#### 2.5.3. Determination of 11 Types of Fatty Acids

The methods for extracting and identifying fatty acids were modified from a previous study conducted by Zhu et al. [26]. After obtaining freeze-dried powder, approximately 500 mg was taken and ground, after which about 50 mg of the powder was weighed. Next, 2 mL of fatty acid extraction solution (chloroform:isopropanol, 2:1) was added, and the sample was placed in the dark at room temperature for 2 h. During this period, a 30 s vortex oscillation was performed every half an hour. After centrifugation for 5 min, 500 μL of the supernatant was taken and transferred to another batch of labeled glass test tubes. Next, 2 mL of sulfuric acid methanol solution was added, the mixture was vortexed and centrifuged at 2500 rpm for 10 s, followed by incubation in an 80 °C water bath for 1 h. After cooling, 2 mL of 0.9% NaCl solution and 1 mL of n-hexane were added, mixed and centrifuged at 2500 rpm for 2 min. Next, the supernatant was transferred to a new glass test tube, and then the residue was washed twice with 2 mL of n-hexane and mixed with the supernatant in the new test tube. After centrifugation (2500 rpm), 700 μL was taken and transferred to an injection bottle, which was placed either directly into the machine or in storage at −20 °C. The fatty acid content was measured via GC-2014 (Shimadzu, Kyoto, Japan) gas chromatography.

#### 2.5.4. Ultra-Performance Liquid Chromatography-Tandem Mass Spectrometry (UPLC-MS/MS) Analysis

UPLC-MS/MS was used to identify the active ingredients in WE. First, 30 mg of ground (30 Hz, 1.5 min) sample powder was weighed, and 1500 μL 70% methanolic aqueous internal standard extract was added. Next, ultrasonic-assisted extraction was conducted. After centrifugation (at a rotation speed of 12,000 rpm for 3 min), the supernatant was obtained, and it was filtered through a microporous membrane (0.22 μm pore size) for subsequent UPLC-MS/MS analysis.

### 2.6. Cell Viability Assay

The human colorectal cancer cell lines SW620 (Procell, Cat.No. CL-0225) and HCT116 (Procell, Cat.No. CL-0096), mouse colon carcinoma CT26.WT (Procell, Cat.No. CL-0072), human breast cancer MCF-7 (Procell, Cat.No. CL-0146), cervical cancer HeLa (Procell, Cat.No. CL-0003), HepG2 (Procell, Cat.No. CL-0107), Huh-7 (Procell, Cat.No. CL-0120) and HK-2 (Procell, Cat.No. CL-0109) were purchased from Wuhan Pricella Biotechnology Co., Ltd. (Procell, Wuhan, China). NCM460 human normal colonic mucosal epithelial cells was purchased from Cyagen Biosciences (OriCell, Cat. No. H1-3701, Guangzhou, China).The cells were cultured in a 37 °C, 5% carbon dioxide incubator. The viability of SW620 and HCT116 cells treated with different fractions for 24 h was analyzed using the CCK-8 assay. The IC50 values were calculated using GraphPad Prism 9.0 software (San Diego, CA, USA). The ZIP synergy score was predicted using the online SynergyFinder Plus (main web-interface: https://synergyfinderplus.org/, mirror used for analysis: https://tangsoftwarelab.shinyapps.io/synergyfinder/, accessed on 7 July 2025).

### 2.7. Analysis of Cell Death and Cell Cycle

SW620 and HCT116 cells were subjected to 24 h treatments and collected. The cell death ratio and cell cycle were examined by using the Annexin V-FITC/PI Apoptosis Detection Kit and cell cycle assay kit, respectively. Both were analyzed using flow cytometry, and data were analyzed using Flow Jo v10 software.

### 2.8. Detection of ROS, GSH and L-LDH

SW620 and HCT116 cells were exposed to various treatments for 24 h and then collected. A flow cytometer was used to analyze the ROS levels. GSH in SW620 and HCT116 cells was detected using GSH kits. For L-LDH detection, cells were treated using the L-LDH assay kit. GSH was detected by measuring absorbance at 405 nm, while L-LDH levels were detected by measuring absorbance at 450 nm using a microplate reader (Scientific Multiskan Sky, Thermo, Waltham, MA, USA).

### 2.9. Cell Proliferation Assay

The proliferation rate of SW620 and HCT116 cells was determined using an EdU assay kit (Beyotime Biotechnology, Nanjing, China). Treated cells were collected, then stained with Alexa Fluor 488 Azide (30 min) and Hoechst 33342 (10 min), and detected using a Keyence BZ-X800E. Cell morphology and fluorescence images were acquired using this microscope; mitochondrial ultrastructure was examined by transmission electron microscopy as described in Section 2.10.

### 2.10. Transmission Electron Microscopy

SW620 and HCT116 cells were subjected to different combinations for 24 h. After treatment, the cells were collected via centrifugation and washed twice with PBS; then, they were fixed overnight at 4 °C in 2.5% glutaraldehyde, washed with PBS twice, and stained with 1% osmium tetroxide for 1 h under gentle rotation. Next, they were rinsed with ddH_2_O three times and fixed with 2% uranyl acetate once for 10 min. Subsequently, the cell samples were dehydrated using 50%, 70%, and 90% ethanol solutions for 15 min each and treated with 100% anhydrous ethanol once for 20 min. Cells were then incubated in a 1:1 mixture of embedder and acetone for two hours, trimmed into ultrathin sections, and imaged using a Talos L120C TEM (FEI, Thermo Fisher Scientific, Waltham, MA, USA).

### 2.11. TUNEL Assay

According to the instructions of TUNEL apoptosis detection reagent, cells were tested with 3 replicates per group. After washing the cells in each well with PBS, briefly, they were fixed with 4% paraformaldehyde for 30 min and washed with PBS twice. Next, the cells were incubated in 0.3% Triton X-100 for 5 min. Then, 100 μL of TUNEL detection solution was added to each group, and they were incubated at 37 °C in the dark for 60 min. After washing with PBS twice, they were stained with Hoechst solution for 10 min. Then, they were washed with PBS once again, and images were obtained using a Keyence BZ-X800E.

### 2.12. Colony Formation Assays

SW620 and HCT116 cells were plated in 6 cm dishes and the medium was replaced every 2 days. The different treatment combinations were carried out for 24h and continued until cell colonies were observed under a microscope. Ultimately, the cells were stained with 0.1% crystal violet.

### 2.13. JC-1 Staining Assay

After the specified treatments, the mitochondrial membrane potential was assessed using an Enhanced Mitochondrial Membrane Potential Assay Kit (Beyotime Biotechnology, C2006). The measurement was performed using a Keyence BZ-X800E in accordance with the manufacturer’s instructions.

### 2.14. Molecular Docking and Dynamics Simulation

The core targets (such as PTGS2) were screened using network pharmacology, and the stability of their binding with active ingredients (such as ALA) was verified via molecular dynamics simulation. The 2D structure of the active ingredients was obtained from the PubChem database and subsequently imported into Chem3D 23.1.1 software to construct and optimize the 3D structure in mol2 format. The crystal structures of the protein targets were obtained by screening high-resolution structures from the RCSB PDB database [27], and redundant groups, such as water molecules and phosphate groups, were removed using PyMOL 2.6 software. After preprocessing, they were saved as PDB format files [28]. The AutoDockTools 1.5.6 was used to perform hydrogenation, dehydration, charge allocation, and rotatable bond definition between proteins and ligands [29], and molecular docking simulations were conducted using AutoDock Vina 1.2.5 software [30]. The coordinates and size of the docking box were set based on the protein activity pocket, and the optimal binding conformation was screened using the binding free energy score. Discovery Studio 2019 and PyMOL 2.6 software were used for visualization and to draw 2D interaction and 3D combination mode diagrams [28].

Based on the molecular docking results, we further investigated the binding between the active ingredients and targets using molecular dynamics simulation experiments. Molecular dynamics simulations of the ALA-PTGS2 complex were performed using GROMACS 2022.2 for 100 ns [31]. In this study, Sobtop_1.0 (dev3.1) software was used to generate a topological file of the GAFF2 parameters [32] based on the ligand structure, and the charge distribution of the ligand was determined using the RESP method to ensure that it conform to the physical and chemical properties. For receptor proteins, the Amber99SB ILDN [33] parameters were used. In the simulation process, the TIP3P water model was used for system solvation, and the minimum distance from protein atoms to the edge of the box was set to 1.0 nm to ensure sufficient solvation of the system. Electrostatic interactions were handled using the particle mesh Ewald (PME) method, and LINCS was employed. The system underwent an energy optimization process: first, 3000 steps of steepest descent optimization were performed, followed by 2000 steps of conjugate gradient optimization. A 100 ns molecular dynamics simulation was conducted at a constant temperature of 310 K and pressure of 1 bar, with a coupling constant of 0.1 ps. During the simulation process, the gmx rmsd, gmx rmsf, gmx hbond, gmx gyre, gmx sasa, and gmx sham tools in GROMACS 2022.2 were used to calculate the root mean square deviation (RMSD), root mean square fluctuation (RMSF), hydrogen bond number, and radius of gyration (Rg). The solvent-accessible surface area (SASA) and free energy landscape map were used to analyze the stability, structural changes, and solvent effects of the system.

### 2.15. Network Pharmacology

The SMILES strings of the major components of WE were retrieved from PubChem (https://pubchem.ncbi.nlm.nih.gov/, accessed on 7 July 2025), and then the Swiss ADME tool (http://www.swissadme.ch, accessed on 7 July 2025) was used to predict the pharmacological properties of the compounds and identify potential core compounds. SwissTargetPrediction (http://www.swisstargetprediction.ch/, accessed on 7 July 2025) was employed to predict the targets of the active compounds. Subsequently, the keyword “colorectal cancer” was used to retrieve relevant targets from the GeneCards database (https://www.genecards.org/, accessed on 7 July 2025) and the OMIM database (https://omim.org, accessed on 7 July 2025), after which the top 2000 targets were further analyzed. Next, duplicates were removed. Targets were imported into the STRING database (https://string-db.org, accessed on 7 July 2025) to generate TSV-format files. The obtained data were analyzed via Cytoscape 3.9.1 to construct a PPI network, and core targets with values above the median were screened. Then, a compound–target–function network was constructed to elucidate the intersection among active ingredients, core targets, and anti-colorectal cancer functions. Components with degree values greater than the median were preliminarily screened for further classification and analyzed.

### 2.16. RNA-Seq Analysis

Total RNA of the different treatment groups was extracted with TRIzol (Invitrogen, Carlsbad, CA, USA) and collected for RNA-seq analysis using the MGISEQ-2000 platform (MGI Tech Co., Ltd., Shenzhen, China). All experiments were performed in triplicate. Differentially expressed genes (DEGs) were identified (*p* < 0.05) using DESeq2 (version 1.4). GO and KEGG were employed for biological processes, cellular components, molecular functions, and pathway enrichment analyses.

### 2.17. qRT-PCR Analysis

The total RNA was extracted from SW620 and HCT116 cells using a Total RNA Isolation Kit (Vazyme Biotech, Nanjing, China). Complementary DNA (cDNA) was obtained with All-in-one RT SuperMix (Applied Biological Materials, Richmond, BC, Canada). RT-PCR was conducted using ChamQ Universal SYBR qRT-PCR Master Mix (Vazyme Biotech) on a LightCycler 96 system (Roche, Basel, Switzerland). Primer sequences are provided in Table S4.

### 2.18. Western Blot Analysis

The total cellular proteins in SW620 and HCT116 cells were extracted with RIPA buffer containing protease and phosphatase inhibitors. Protein concentrations were quantified using a BCA kit. Equal amounts of protein were separated via SDS-PAGE and transferred to PVDF membranes. The membranes were blocked with 5% non-fat milk or BSA (for phosphorylated proteins) and then incubated overnight at 4 °C with primary antibodies against Bcl-2 (Proteintech Group, Wuhan, China, Cat No. 12789-1-AP), BAX (Proteintech Group, Wuhan, China, Cat No. 50599-2-Ig), caspase-9 (Proteintech Group, Wuhan, China, Cat No. 10380-1-AP), caspase-3 (Proteintech Group, Wuhan, China, Cat No. 19677-1-AP), and p53 (Proteintech Group, Wuhan, China, Cat No. 10442-1-AP), followed by HRP-conjugated secondary antibodies. Protein bands were visualized using ECL reagents. β-actin (Proteintech Group, Wuhan, China, Cat No. 20536-1-AP) was used as the internal reference. All experiments were performed in triplicate.

### 2.19. Safety Assessment

#### 2.19.1. Animals

Sprague Dawley rats (female, 6 weeks old, and weighing around 160–180 g, SPF) were purchased from Zhejiang Vital River Laboratory Animal Technology Co., Ltd. (Hangzhou, China). The animals were maintained in polypropylene cages under regulated environmental parameters (temperature: 25 °C ± 2 °C; 12 h light/dark cycle; relative humidity: 50–60%), with free access to food and water during the acclimatization period before the experiment. All experimental procedures were conducted in accordance with the guidelines established by the Ethical Committee of the ZJU-Laboratory Animal Welfare and Ethics Review under ethical approval number ZJU20260459.

#### 2.19.2. Acute Toxicity

An acute oral toxicity study was performed according to protocol 425 of the Organization for Economic Cooperation and Development (OECD). A limit dose of 2000 mg/kg of the WE was administered to female rats (*n* = 3) via oral gavage after a 6 h fast; 0.9% saline solution was used in the control group. General behavioral changes, clinical symptoms, and mortality of the rats were continuously observed and recorded for 24 h after administration. Thereafter, the animals were observed daily for 14 days. Water consumption, food intake, and body weight changes in the rats were measured and recorded once a day until the experimental endpoint. All rats were anesthetized with isoflurane, and their organs (the heart, liver, spleen, lungs, and kidneys) were collected.

#### 2.19.3. Hematological and Biochemical Analyses

Blood samples were collected from Sprague Dawley rats after the 14-day acute toxicity observation period. Whole blood samples were subjected to a routine hematological analysis using a Mindray BC-5000 automated hematology analyzer (Mindray, Shenzhen, China) to measure white blood cell count, red blood cell count, platelet count, and other hematological parameters. Serum was separated via centrifugation for subsequent biochemical detection. Serum levels of total bilirubin (TBIL), creatinine (CREA), total protein (TP), alanine aminotransferase (ALT), aspartate aminotransferase (AST), and other liver and kidney function indices were determined using an automatic biochemical analyzer.

#### 2.19.4. Macroscopic and Histological Analyses

Tissue samples were harvested at the end of the experiment. Gross morphological alterations in the tissues were carefully evaluated in terms of weight and appearance (macroscopic analysis).

For histological examination, specimens were fixed in 4% paraformaldehyde, dehydrated with gradient ethanol, cleared in xylene, embedded in paraffin wax, and then stained with hematoxylin and eosin (HE). Finally, they were assessed and photographed for further analysis.

### 2.20. Statistical Analysis

Data were analyzed using GraphPad Prism 9.0 software (GraphPad Software, San Diego, CA, USA). Experiments were performed in triplicate, and results are presented as mean ± standard deviation (SD). The differences between two groups were assessed using the *t*-test and one-way analysis of variance (ANOVA) followed by Dunnett’s multiple comparison test. Statistical significance was set as follows: *p* * < 0.05, *p* ** < 0.01, and *p* *** < 0.001.

## 3. Results

### 3.1. Outline of the Experimental Design

**Figure 1:**
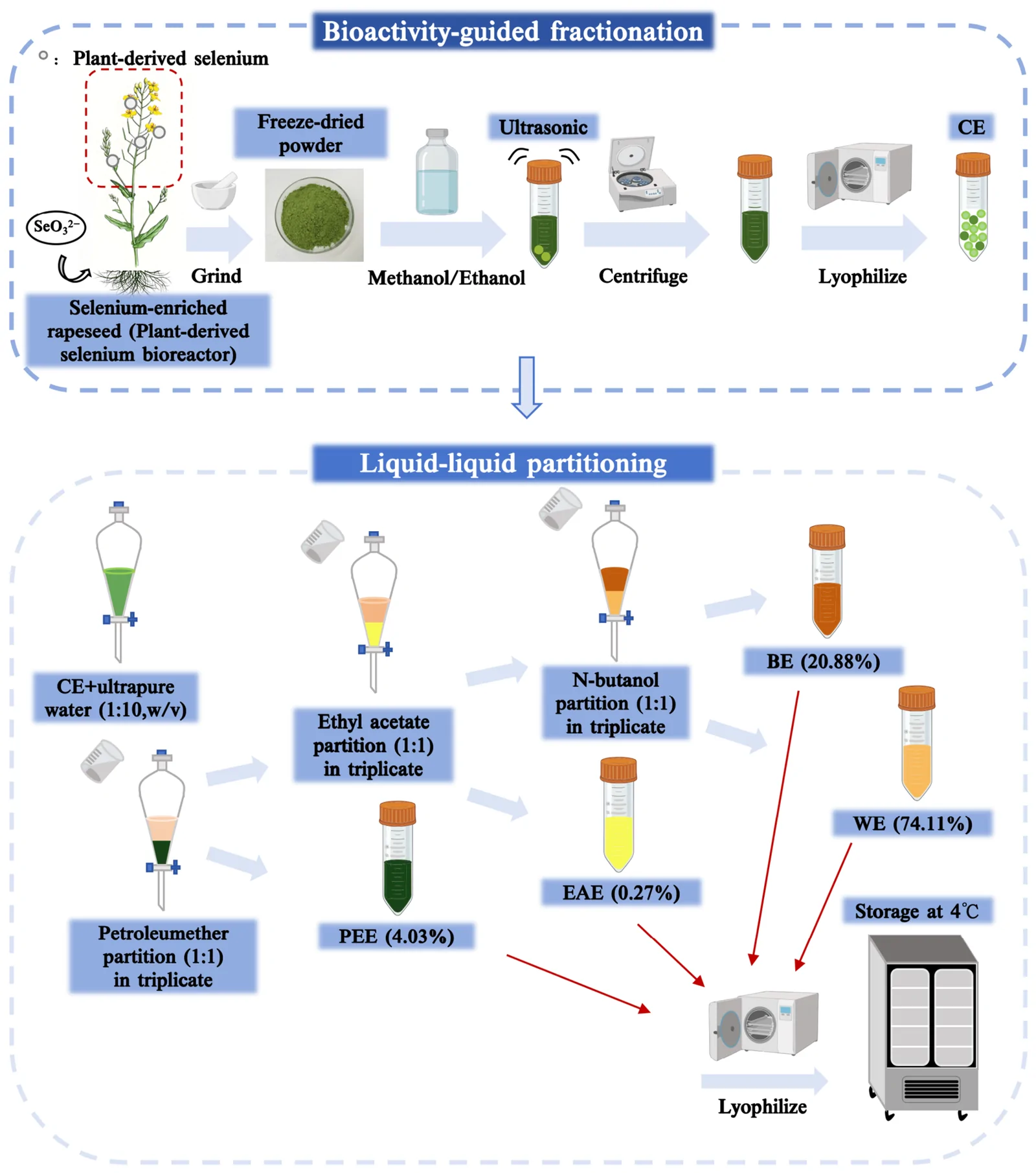
Flowchart of crude extract preparation and subsequent fractionation using different solvents. Arrows indicate the sequence of extraction, liquid–liquid partitioning, lyophilization, and storage steps.

Figure 1 presents a flowchart summarizing the overall experimental design described in this study. First, the fresh inflorescences of selenium-enriched rapeseed were harvested and ground into powders, and then placed in a freeze dryer for approximately 72 h. After lyophilization, dry powder was extracted with different concentrations of methanol or ethanol, and the products obtained were designated crude extracts (CEs). The CEs were assessed in terms of their effects on the different cancer cells alone or in combination with CDDP. The most effective CE was lyophilized, and the resulting powder was dissolved in distilled water. The aqueous solution was further sequentially extracted with PEE, EAE, and BE. The solution remaining after PEE, EAE and BE extraction was assigned as WE. The PEE, EAE and BE extraction fractions and remaining WE were assessed in terms of their effects on the cancer cells, via acting alone or in combination with CDDP.

### 3.2. The 50% Ethanol Extracts Were More Effective in Inhibiting Cancer Cell Growth Either Alone or in Combination with CDDP

**Figure 2:**
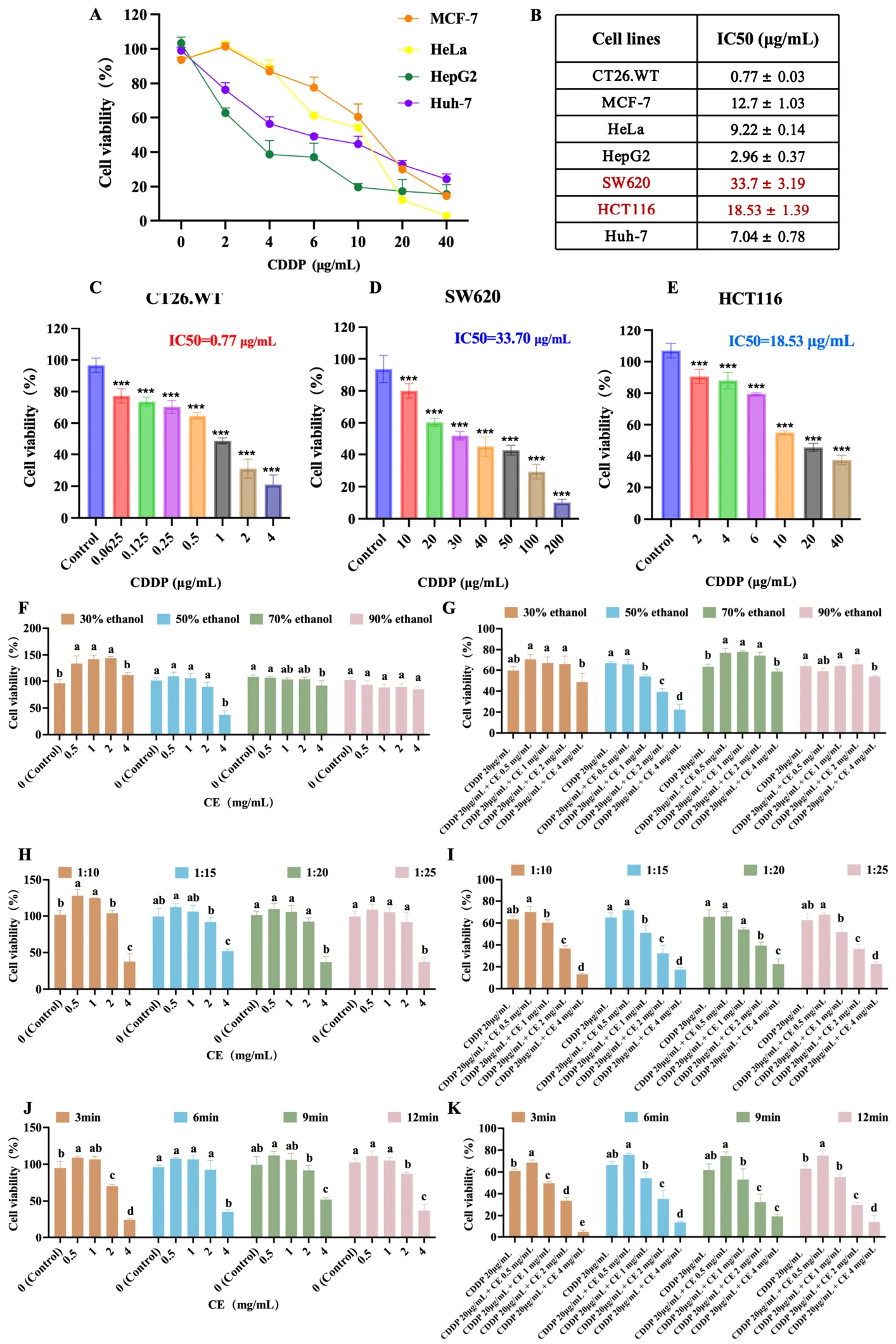
CE significantly enhanced the sensitivity of cancer cells to CDDP. (**A**) Cancer cell viability was evaluated via the CCK-8 assay after 24 h of treatment with different concentrations of CDDP. Control: Cells without CDDP treatment. (**B**) IC50 values of CDDP calculated from the data in (**A**,**C**–**E**). (**C**–**E**) Viabilities of CT26.WT, SW620 and HCT116 cells upon CDDP treatment. (**F**,**G**) Effects of ethanol concentration on the viability of SW620 cells treated with CE alone or co-treated with CE and CDDP. (**H**,**I**) Effects of the solid–liquid ratio on the viability of SW620 cells treated with CE alone or co-treated with CE and CDDP. (**J**,**K**) Effects of extraction time on the viability of SW620 cells treated with CE alone or co-treated with CE and CDDP. Data are expressed as mean ± SD (*n* = 3). *** *p* < 0.001 versus the control group. Different lowercase letters (a, b, c, d) denote significant differences within each color-coded group (*p* < 0.05). Red in (**B**) identifies the selected CRC cell lines.

To investigate the sensitivity of cancer cells to CDDP, a CCK-8 assay was conducted to assess the growth of various cell lines derived from humans (MCF-7, HCT116, SW620, HeLa, HepG2, and Huh-7) or mice (CT26.WT) following 24h treatment with CDDP at different concentrations. Although this study mainly focuses on the anti-CRC activity of the extract, we also evaluated its effects in several non-colorectal cell lines. Compared with the control (without the addition of CDDP), the CDDP treatment clearly inhibited the growth of all tested cell lines in a dose-dependent manner, with IC50 values ranging from 0.77 μg/mL to 33.70 μg/mL (Figure 2A,B).

We found that, in general, the human cancer cell lines exhibited stronger drug resistance than the mouse cancer cell line (Figure 2A,C–E). Data obtained from multiple cell lines serve only as a preliminary potency screening and provide a theoretical basis for our follow-up CRC-focused investigation. We also found that the CRC cell lines SW620 and HCT116 exhibited stronger resistance to CDDP than the other cancer cell lines (Figure 2B). We therefore chose SW620, which showed the highest IC50 value, as the cell model to determine which selenium-enriched rapeseed extraction could enhance the effect of CDDP. As the half-maximal inhibitory concentration (IC50) of CDDP for SW620 cells was 33.70 μg/mL (Figure 2D), we decided to use 20 μg/mL CDDP in subsequent experiments.

Given that the selection of extraction solvent, solvent concentration, solid–liquid ratio and extraction time is crucial for ultrasonic extraction of bioactive compounds from natural products, we assessed the impact of the extracts obtained from methanol and ethanol extraction with concentrations ranging from 30% to 90% on cell viability, using them either acting alone or in combination with CDDP. As shown in Figure 2F–G and Figure S1, regardless of whether methanol or ethanol was used as the solvent, the extract alone showed inhibitory effects on SW620 cell proliferation at higher concentrations. Nevertheless, we observed that the 50% ethanol and 30% methanol extracts could enhance the effect of CDDP at lower concentrations. Because we noticed that the 30% methanol extract alone appeared to promote the growth of SW620 cells at low dosages, we chose 50% ethanol as the optimal extraction solvent for subsequent experiments.

As illustrated in Figure 2H,I, the 50% ethanol extract obtained with a solid–liquid ratio of 1:10 (the weight of the powder versus the liquid volume) promoted cancer cell growth at concentrations of 0.5 and 1.0 mg/mL. In contrast, when applied alone, the extracts obtained using a solid–liquid ratio of 1:15 and 1:20 at concentrations of 0.5, 1.0 and 2.0 mg/mL exerted almost no effect on cell viability, but showed a significant growth inhibitory effect at 4 mg/mL. Importantly, we found that, when used in combination with CDDP, the extracts at concentrations of 2.0 and 4.0 mg/mL significantly enhanced the sensitivity of cancer cells to CDDP. Taken together, considering both cost and cell activity, we determined that a solid-to-liquid ratio of 1:15 was the optimum ratio and used the extracts obtained with this ratio for further experiments.

Next, the effects of extracts obtained using different ultrasonic durations (3–12 min) on cell viability were studied. Figure 2J,K show that, with 6 min sonication, extracts with a concentration in the range of 2–4 mg/mL significantly enhanced the toxicity of CDDP to SW620 cells. To reduce extraction time and ensure the effectiveness of the treatment combination on cell viability, the 6-min sonication duration was chosen in the following experiments.

### 3.3. WE Fraction Contains Bioactive Compounds for Inhibiting the Growth of CRC Cells

**Figure 3:**
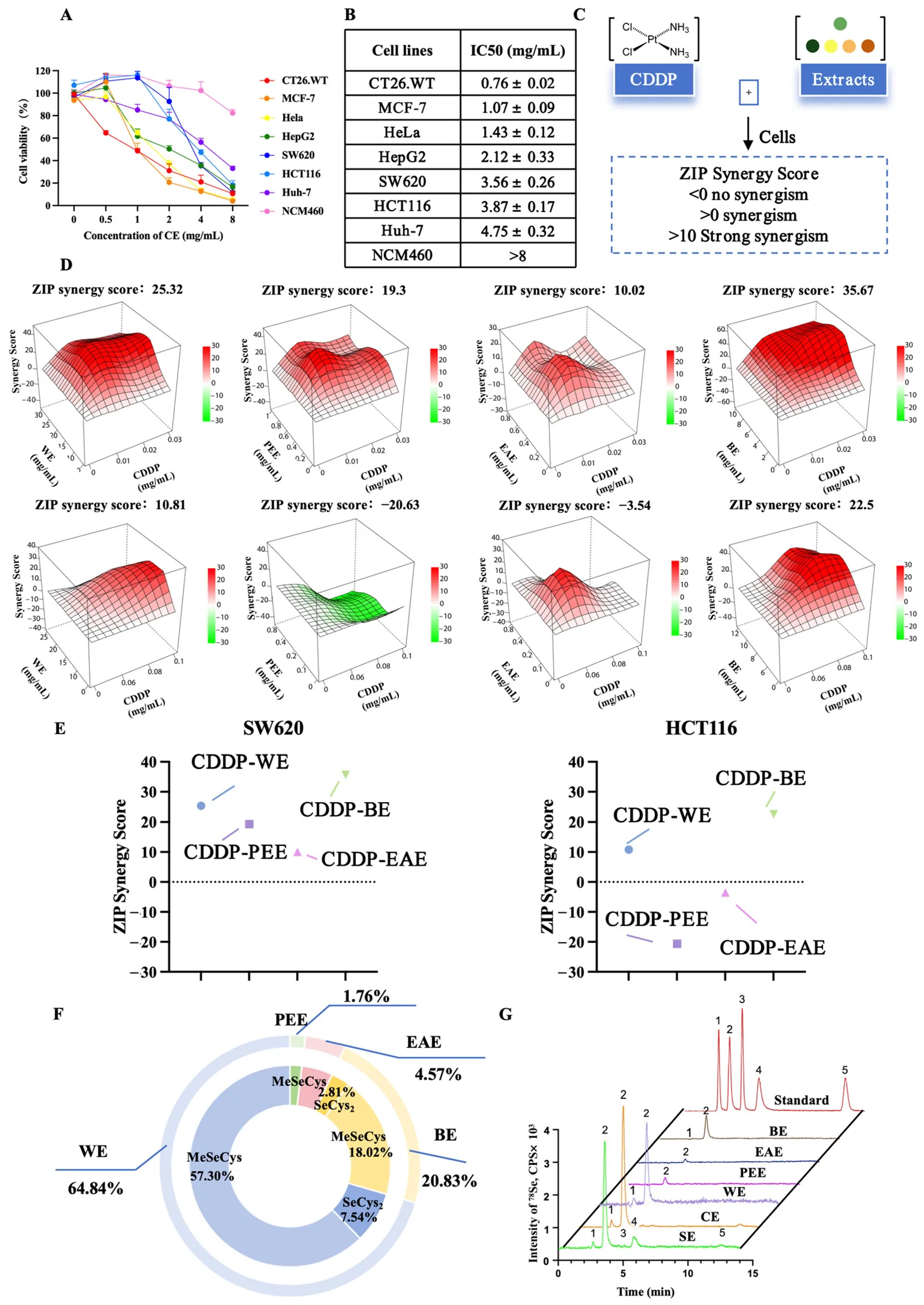
Evaluation of the effects of synergies between different extracts and CDDP on their abilities to fight against cancer cells in vitro. (**A**) The CCK-8 assays assess the effect of the water-redissolved lyophilized 50% ethanol CE alone on the proliferation of different types of cancer cells and the cytotoxicity of colon mucosal epithelial cell line NCM460 treated for 24 h at the indicated concentrations. (**B**) IC50 values of CE in CRC cells and NCM460 cells as treated in (**A**). (**C**) Study design: SW620 and HCT116 cells were treated with CDDP and WE for drug sensitivity. (**D**,**E**) The synergistic effects were quantified using the ZIP synergy scores (ZIP synergy scores greater than 0 were considered indicative of synergism, and scores above 10 were considered strongly synergistic) via the SynergyFinder Plus web application, based on the cell viability from different extract combinations. (**F**) Analysis of total selenium content in PEE, EAE, BE, and WE. (**G**) HPLC chromatograms of five selenium species in standard solutions, CE, PEE, EAE, BE, and WE. Peaks 1–5 in the chromatograms correspond to SeCys2, MSC, Se (IV), SeMet, and Se (VI), respectively. In (**E**), the dashed line denotes a ZIP score of 0. In (**F**), colors distinguish the labeled extraction fractions and selenium species. In (**G**), colors distinguish the labeled samples.

We first assayed the effect of the CE on the growth of seven types of cancer cells (CT26.WT, MCF-7, HCT116, SW620, HeLa, HepG2, and Huh-7) and a human colon mucosal epithelial cell line (NCM460) (Figure 3A). The IC50 values of each type of cell are presented in Figure 3B. To further define the active ingredients, the lyophilized 50% ethanol extracts were dissolved in distilled water (1:10 *w*/*v*). We prepared four different extraction fractions according to Figure 1, namely PEE, EAE, BE, and the remaining water-redissolved CE (named the WE). We then assessed the possible synergistic effects of these fractions in combination with CDDP on the growth of SW620 and HCT116 cells using a CCK-8 assay. We quantified the synergistic effects using ZIP synergy scores. A ZIP synergy score >0 indicates synergy, and a score >10 indicates strong synergy, as calculated using SynergyFinder Plus web application [34] (Figure 3C). We observed that, when all extraction fractions were used alone, they showed inhibitory effects at higher concentrations; however, when they were used in combination with CDDP, they significantly enhanced the inhibitory effect on the proliferation of SW620 and HCT116 cells. Specifically, a notable synergistic inhibitory effect was exhibited by combinations such as CDDP-WE (ZIP: 25.32), CDDP-PEE (ZIP: 19.3), CDDP-EAE (ZIP: 10.02) and CDDP-BE (ZIP: 35.67) on SW620 cells and by CDDP-WE (ZIP: 10.81) and CDDP-BE (ZIP: 22.5) on HCT116 cells, while CDDP-PEE (ZIP: −20.63) and CDDP-EAE (ZIP: −3.54) did not show a synergistic effect on HCT116 cells (Figure 3D,E).

We then analyzed the total selenium contents in different fractions by subjecting the acid-digested fractions to ICP-MS. As shown in Table S1, the WE fraction accounted for 64.84% of the total selenium in the CE. Next, we investigated the chemical speciation of selenium in the fractions (CE, PEE, EAE, BE, and WE). The HPLC-ICP-MS results identified five selenium species in the fractions (Figure 3F,G). Interestingly, neither SeCys nor Se (VI) was detected in any fraction. SeMet was only detected in the CE. These results showed that MSC was the predominant selenium species in the CE and was largely retained in the WE (74.11%). Eleven fatty acids of SE were identified via GC (Table S2). Taking into account the selenium compositions and the effect on SW620 and HCT116 cells (Figure S2 and Table S1), the WE was selected for the subsequent experiments. The CDDP-WE combination showed a significant synergistic effect compared to their individual use. In summary, the CDDP-WE combination is considered a combinatorial strategy with anti-colorectal cancer potential.

### 3.4. WE Enhances CDDP-Induced Oxidative Stress in CRC Cells

**Figure 4:**
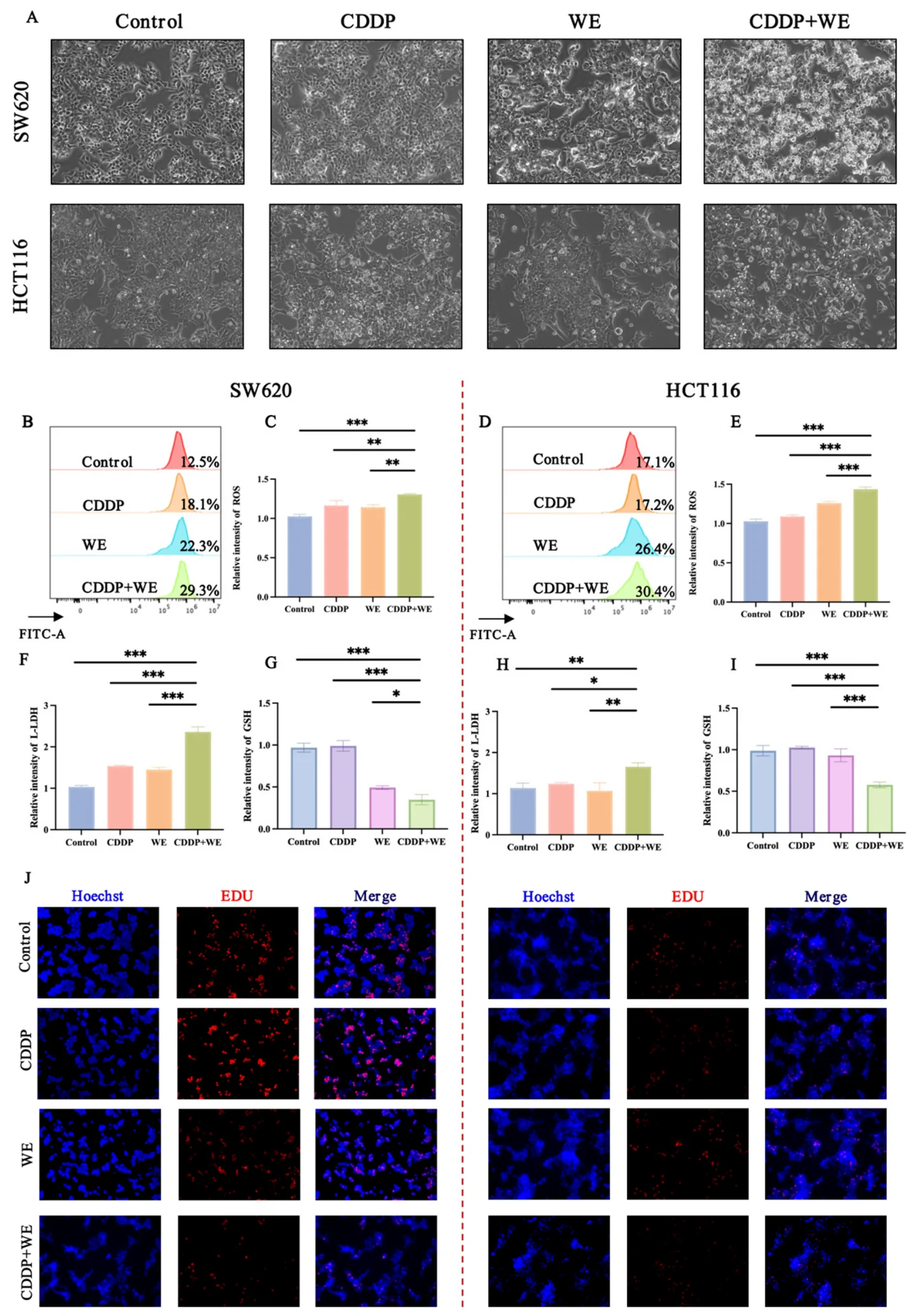
The CDDP-WE combination treatment synergistically increases oxidative stress and induces cell death. (**A**) Cell morphology of the SW620 and HCT116 cells after the CDDP-WE combination treatment for 24 h. (**B**–**E**) Measurement of the ROS release. (**F**,**H**) Measurement of the LDH release. (**G**,**I**) Measurement of intracellular GSH levels. (**J**) Fluorescent staining for the EdU assay. The mean ± SD is shown, with *n* = 3. * *p* < 0.05, ** *p* < 0.01, and *** *p* < 0.001. In (**J**), blue fluorescence labels nuclei stained with Hoechst, and red fluorescence labels EdU-positive cells; merged images show both channels.

To assess the effects of the CDDP-WE combination on SW620 and HCT116 cells, we observed the cell morphological changes caused by different treatments (Figure 4A). The CDDP-WE combination treatment reduced cell proliferation and increased the proportion of cell debris, suggesting combinatory antiproliferative and cytotoxic effects. To further investigate the mechanism of action of the CDDP-WE combination on CRC cells, we measured the ROS levels after CDDP-WE treatment. Compared with the WE treatment alone, the ROS levels in SW620 and HCT116 cells were elevated significantly after the CDDP-WE combination treatment (Figure 4B–E). These results suggest that the CDDP-WE combination inhibits CRC cell growth by promoting ROS accumulation. A lactate dehydrogenase (LDH) release assay revealed a notable increase in LDH levels in the CDDP-WE combination group, indicating cell membrane rupture and CRC cell death (Figure 4F,H). The addition of the WE led to the depletion of GSH in cancer cells, exacerbated oxidative stress damage induced by CDDP, and improved the therapeutic effect of CDDP (Figure 4G,I). Moreover, our results suggest that the EC50 obtained from the ABTS assay can be used to evaluate the extracellular antioxidant activity of WE, demonstrating its favorable free radical scavenging capacity. (Figure S3) [32].

### 3.5. The CDDP-WE Combination Treatment Inhibits CRC Cell Proliferation and Promotes Cell Apoptosis

**Figure 5:**
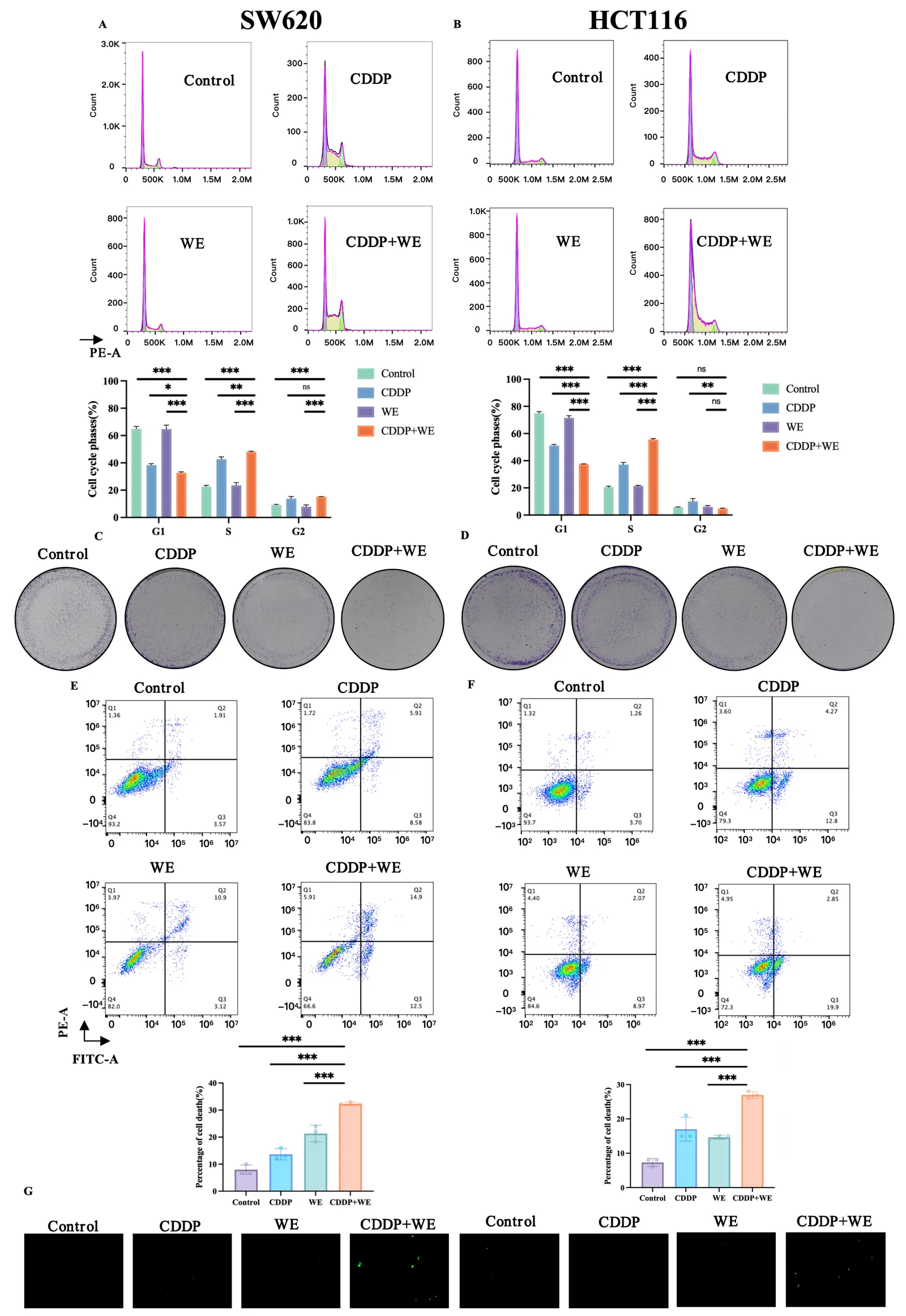
The CDDP and WE combination treatment synergistically inhibits cell proliferation and triggers apoptosis in CRC cells. (**A**,**B**) CDDP-WE influenced the cell cycle in SW620 and HCT116 cells; the cell cycle was analyzed by PI staining and detected using flow cytometry. (**C**,**D**) Colony formation after the CDDP and WE combination treatment for 2 weeks in SW620 and HCT116 cells. The colonies were fixed in methanol and stained with crystal violet. (**E**,**F**) Cell death rate was assessed by flow cytometry after staining with the phartmingen Annexin V-FITC/PI Apoptosis Detection Kit in SW620 and HCT116 cells following CDDP and WE treatment for 24 h. (**G**) Fluorescent images obtained using a fluorescence microscope. Apoptosis-induced cells released green fluorescence upon analysis with the DeadEnd™ Fluorometric TUNEL System. The mean ± SD is presented, with *n* = 3. * *p* < 0.05, ** *p* < 0.01, and *** *p* < 0.001 versus the indicated comparison groups. In the cell-cycle bar charts, colors distinguish the different periods identified in the legends.

An EdU labeling assay revealed that the CDDP-WE combination exerted a significant synergistic inhibitory effect on cell proliferation (Figure 4J). Treating SW620 and HCT116 cells with the CDDP-WE combination shifted the distribution of cells from the G0/G1 phase to the S and G2/M phases, as revealed by flow cytometry analysis. The proportion of S-phase cells in the CDDP-WE treatment groups notably increased, whereas the WE treatment alone did not induce any changes in the cell cycle phases (Figure 5A,B). The results indicate that the WE sensitized the CRC cells to CDDP through cell cycle arrest in the S phase, thereby inhibiting cell proliferation. To further evaluate the antiproliferative effects, colony formation assays were performed. The CDDP-WE treatment significantly reduced colony formation compared with the individual treatments, and this effect was significantly stronger than that of the CDDP treatment alone (Figure 5C,D).

Next, we attempted to investigate the anticancer function of the CDDP-WE combination by analyzing cell viability. We adopted flow cytometry to analyze cell death through Annexin V/PI double staining. The percentages of apoptotic SW620 and HCT116 cells significantly increased after incubation with the CDDP-WE combination, reaching 34% and 28%, respectively (Figure 5E,F).

The TUNEL staining results showed that there were very few apoptotic cells in the control group, while the fluorescence intensity of apoptosis significantly increased in the CDDP-WE groups. A large number of green apoptotic cells was observed in the fluorescence images (Figure 5G), suggesting that, in addition to arresting cell proliferation, the CDDP-WE combination also effectively promotes apoptosis.

Taken together, the roles of the CDDP-WE combination in promoting ROS production, inhibiting cell proliferation and triggering cell apoptosis are likely the main factors contributing to its anti-CRC effect.

### 3.6. CDDP-WE Treatment Synergistically Induces Apoptosis Through Mitochondrial-Dependent Pathway

**Figure 6:**
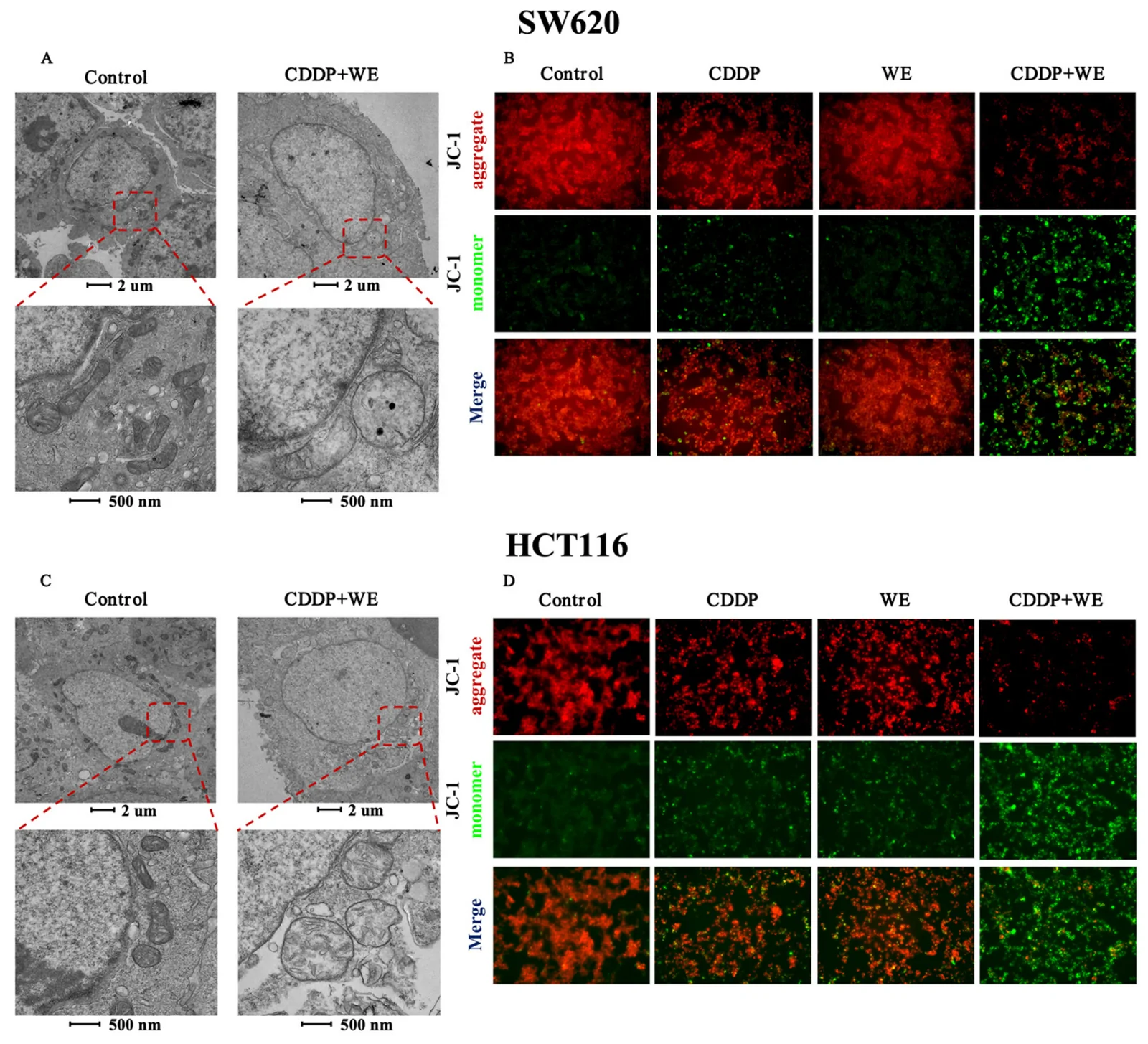
The CDDP-WE combination treatment activates the mitochondrial intrinsic apoptotic pathway. (**A**,**C**) TEM observation of the mitochondrial morphology. (**B**,**D**) Membrane potential detection of the CRC cells. Red JC-1 aggregate fluorescence indicates polarized mitochondria, whereas green JC-1 monomer fluorescence indicates reduced mitochondrial membrane potential; merged images combine both channels.

To investigate whether mitochondrial dysfunction contributed to CDDP-WE-induced apoptosis, the SW620 and HCT116 cells were analyzed via transmission electron microscopy (TEM). TEM showed that, compared with the control groups, the CDDP-WE combination-treated SW620 and HCT116 cells exhibited a deformed mitochondrial morphology, such as mitochondrial cristae ambiguity and swelling (Figure 6A,C). An increasing number of studies have indicated that the breakdown of MMP (mitochondrial membrane potential) may lead to mitochondrial dysfunction, which plays a dual role in natural product-induced cell apoptosis [35]. To investigate whether the CDDP-WE combination treatment-induced cell apoptosis occurred through the mitochondrial apoptosis pathway, a JC-1 kit was used to detect changes in MMP in SW620 and HCT116 cells. Green fluorescence marked cells that had undergone apoptosis or necrosis, while red fluorescence marked alive cells that maintained normal mitochondrial membrane potential. As shown in Figure 6B,D, compared with the control group, the red fluorescence was significantly reduced and green fluorescence was enhanced in the CDDP-WE group, indicating a decrease in mitochondrial membrane potential coupled with an increase in apoptotic cells. Therefore, it appears that the CDDP-WE combination synergistically activates apoptosis through the intrinsic mitochondrial apoptotic pathway in CRC cells.

### 3.7. Characterization of the Chemical Profile of Selenium-Enriched Rapeseed

**Figure 7:**
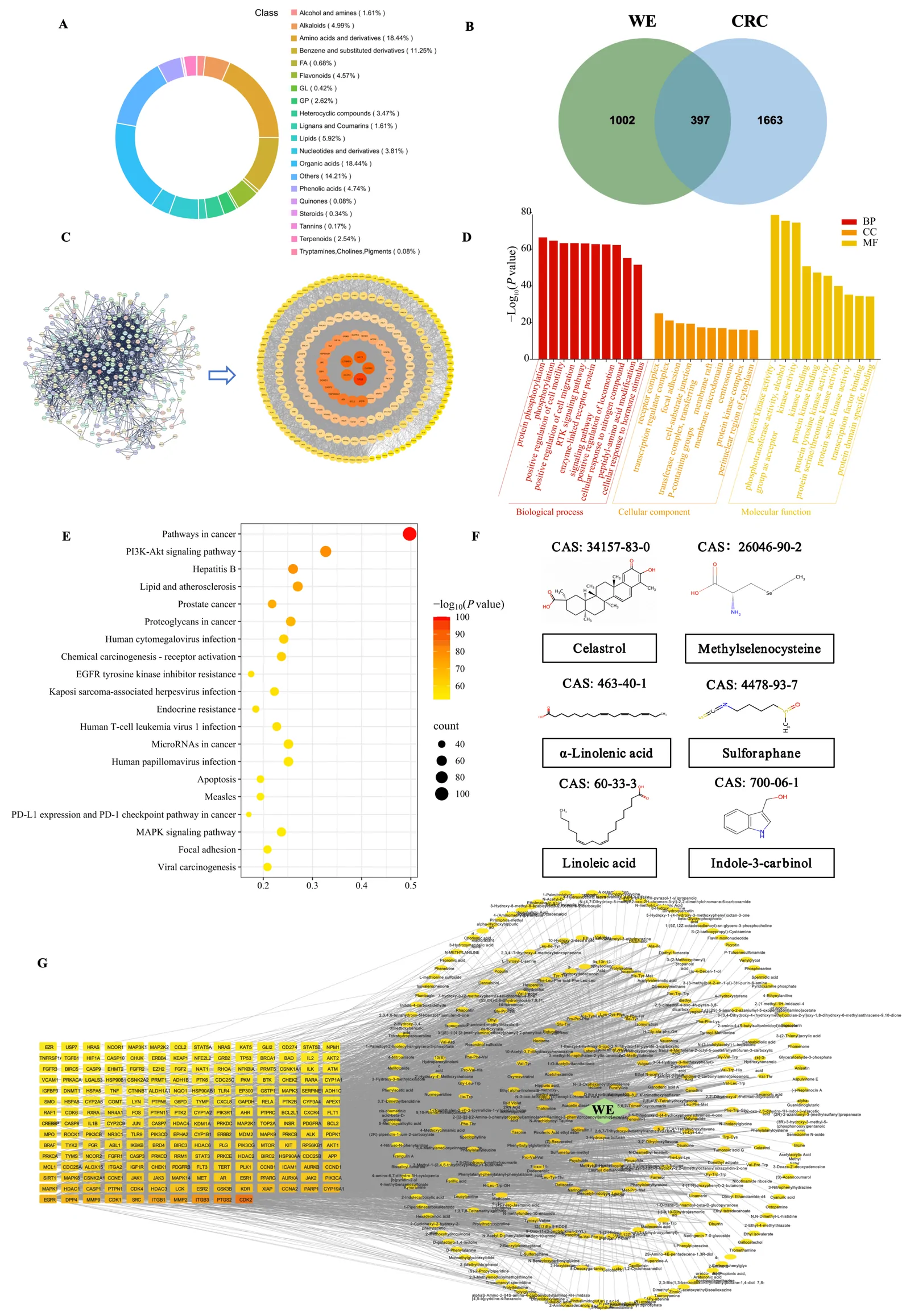
Network pharmacology analysis of WE in the treatment of colorectal cancer. (**A**) Structural classification of compounds contained in the WE. (**B**) Venn diagram of gene sets between groups. (**C**) PPI network. (**D**–**E**) GO and KEGG pathway enrichment analyses of DEGs. (**F**) Chemical structures of the compounds that are considered as the basic active ingredients with anti-colorectal cancer potential. (**G**) Network of drug active components–intersection targets.

Next, we comprehensively analyzed the chemical composition of the WE using ultra-performance liquid chromatography–tandem mass spectrometry (UPLC-MS/MS). Selenium-enriched rapeseed is a classical natural product and a source of active ingredients with anticancer effects. As shown in Figure S4, the primary chromatograms of the WE were generated in both positive and negative ion modes. The molecular formulas and chemical profile of the WE were further analyzed through reference databases and tandem mass spectrometry interpretation. Through prediction, a total of 314 high-content chemical compounds were identified in the WE, including the following classes: 76 amino acids and derivatives, 46 benzenes and substituted derivatives, 36 organic acids, 32 heterocyclic compounds, 29 others, 16 alcohols and amines, 16 alkaloids, 13 lipids, 12 phenolic acids, 11 flavonoids, 7 terpenoids, 6 fatty acids (FAs), 4 Glycerophospholipids (GPs), 4 nucleotides and derivatives, 4 lignans and coumarins, 1 quinone and 1 steroid (Figure 7A). The metabolome demonstrates the diversity and complexity of the WE; thus, it is deemed necessary to explore the main active ingredients in the WE that synergistically exert anti-CRC effects with chemotherapeutics.

### 3.8. Network Pharmacology Analysis of the Main Active Ingredients of WE in the Treatment of CRC

Network pharmacology has become a key approach for exploring the core targets of natural products. A total of 327 compounds, comprising two selenium-containing compounds identified via HPLC-ICP-MS, 314 non-selenium compounds determined via UPLC-MS/MS, and 11 fatty acids detected via GC, exhibiting bioavailability and drug-likeness were imported into the SwissTargetPrediction database to predict their potential targets. A total of 1399 compound–target interactions were identified. The keyword “colorectal cancer” was searched in the GeneCards database to identify relevant targets, resulting in 125,003 targets. After sorting them by score value and selecting the top 2000 targets for further analysis, we obtained 96 targets from the OMIM database. Consequently, we analyzed the intersection of targets and obtained 2060 targets after deduplication (Figure 7B). After setting the confidence score to 0.900 (high confidence), removing free nodes, and screening, we acquired 397 intersecting targets, which were subsequently imported into the STRING database. Then, a protein–protein interaction (PPI) network was obtained and further analyzed and visualized using the CentiScaPe plug-in within Cytoscape 3.7.1. Three centrality measures (degree, closeness, and betweenness centrality) were calculated for each target. Then, the intersection was taken, and 206 core targets (whose values exceeded the corresponding medians) were obtained (Figure 7C). The targets identified included CDK2, PTGS2, ITGB3, matrix metalloproteinase 3 (MMP3), ITGB1, SRC, CDK1, MMP9, DPP4, and EGFR, which emerged as the top ten core targets, and these nodes represented the key targets of the WE against CRC cells. In addition, the above 206 core targets were imported into the DAVID database for GO and KEGG analysis. The pathways enriched by KEGG included the PI3K-Akt, apoptosis, and MAPK signaling pathways and pathways in cancer cells that were associated with cell apoptosis. The GO enrichment analysis showed that the biological processes involved in the WE treatment of colorectal cancer were mainly enriched in the regulation of protein phosphorylation and the positive regulation of cell motility and migration. The cellular components mainly included receptor complex and transcription regulator complex; the molecular functions mainly focused on kinase activity, protein kinase binding, and transcription factor binding (Figure 7D,E). A review of the literature revealed that Cel, MSC, ALA, SFN, LA, and I3C have been used as synergistic chemotherapy drugs to enhance anti-CRC efficacy. Therefore, they were selected as the active ingredients (ligands) for molecular docking (Figure 7F). To elucidate correlations between functional active ingredients in the WE and the core targets linked to CRC, a compound–target–function network was constructed (Figure 7G).

**Figure 8:**
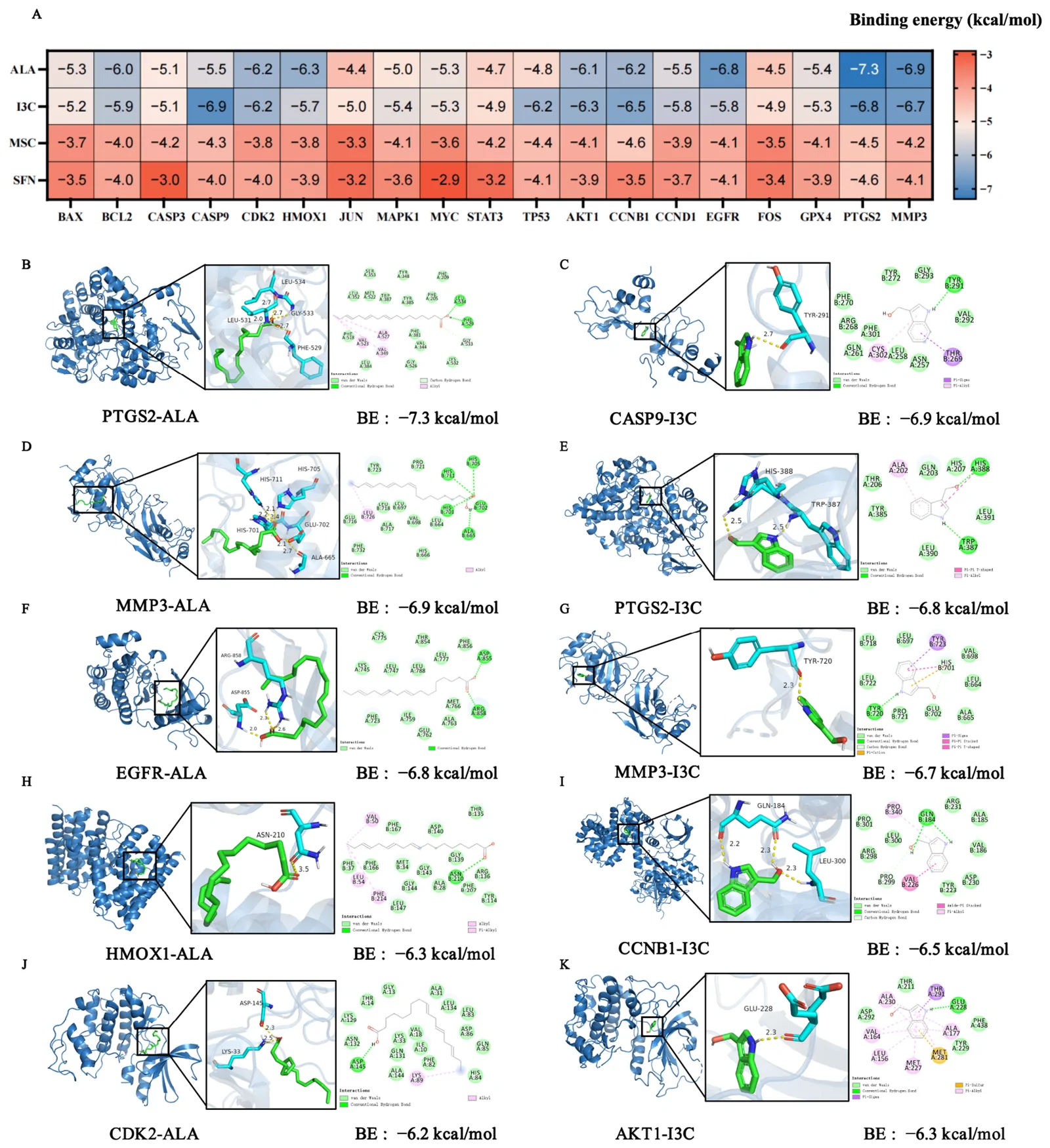
Molecular docking simulation of ALA and I3C interactions with core colorectal cancer-associated targets. (**A**) Binding affinity heatmap of ALA and I3C against 19 core targets, color-scaled by docking energy (kcal/mol). (**B**) ALA-PTGS2 protein molecular docking. (**C**) I3C-CASP9 protein molecular docking. (**D**) ALA-MMP3 protein molecular docking. (**E**) I3C-PTGS2 protein molecular docking. (**F**) ALA-EGFR protein molecular docking. (**G**) I3C-MMP3 protein molecular docking. (**H**) ALA-HMOX1 protein molecular docking. (**I**) I3C-CCNB1 protein molecular docking. (**J**) ALA-CDK2 protein molecular docking. (**K**) I3C-AKT1 protein molecular docking. In (**A**), the color scale represents docking binding energy, with blue indicating more negative values and red indicating less negative values, respectively.

BAX, BCL2, CASP3, CASP9, CDK2, HMOX1, JUN, MAPK1, MYC, STAT3, TP53, AKT1, CCNB1, CCND1, EGFR, FOS, GPX4, PTGS2 and MMP3 appeared more frequently in the KEGG-enriched pathway and are known to be associated with cell growth and development (Figure 8A). In addition, some of them are related to cellular oxidative stress. Therefore, these nineteen proteins were selected as protein targets for the docking of the ligands.

### 3.9. Molecular Docking and Molecular Dynamics Simulations Reveal Stable Interactions Between Active Ingredients and Core Therapeutic Targets

A docking energy value of <−4.25 kcal/mol (1 cal ≈ 4.186 J) is generally acceptable for indicating binding activity between molecules, whereas <−5.0 kcal/mol and <−7.0 kcal/mol indicate good and strong binding activity, respectively. Based on the network pharmacology enrichment results, we conducted a molecular docking analysis of ligands and targets to evaluate their affinities (Figure 8A and Table S5). Molecular docking and structural visualization showed that ALA and I3C had favorable binding affinities toward the target proteins in Figure 8B–K. Among the components, ALA exhibited the strongest binding ability with PTGS2. The binding score between ALA and PTGS2 was below −7 kcal/mol. The Leu 534 and Phe 529 residues on the PTGS2 receptor formed hydrogen bonding interactions with ALA, while the Ala 527, Val 523, and Val 349 residues on the PTGS2 receptor formed hydrophobic interactions with ALA. Ser 353, Tyr 348, Phe 209, Phe 205, Tyr 385, Trp 387, Met 522, Leu 352, Phe 518, Leu 384, Phe 381, Val 344, Gly 526 and Lys 532 residues formed a van der Waals interactions with ALA.

Further analysis based on the solvent-accessible surface area (SASA), radius of gyration (Rg), and free energy landscapes indicated that ALA-PTGS2 exhibited relatively stable fluctuations during its movement (Figure S5A,B) and that ALA-PTGS2 did not undergo significant expansion or contraction. After binding, there was no significant change in the SASA of the complex, further demonstrating that ALA-PTGS2 could achieve relatively stable binding (Figure S5C). Hydrogen bonds play an important role in the binding of ligands to proteins. The number of hydrogen bonds between ALA and PTGS2 during the dynamic process is shown in Figure S5D. The number of hydrogen bonds between small molecules and target proteins ranged from 0 to 3, and, in most cases, ALA-PTGS2 had approximately two hydrogen bonds. This indicates that ALA has good hydrogen bonding interactions with PTGS2. The root mean square fluctuation (RMSF) can indicate the flexibility of amino acid residues in proteins. As shown in Figure S5E, the RMSF value of this complex was relatively low (mostly below 0.4 nm), resulting in low flexibility and high stability. The free energy landscape (FEL) is shown in Figure S5F, which also shows the surface free energy results of ALA-PTGS2; the lower free energy indicates that these conformations are more stable. Subsequently, using the binding conformation of the complex, the binding free energy between the small molecule and the target protein was calculated using the MM/PBSA method (Figure S6A). The binding free energy of the complex system was −29.14 kcal/mol. The contribution of key binding residues to complex stability was further calculated by analyzing residue interaction fractions. The results show that the residues Leu 531, Ala 527, Leu 534, Asn 375, Val 116, Val 349, Gly 533 and Ile 377 had high contribution values in the complex system (Figure S6B,C). This suggests that these amino acid residues may play an important role in the catalytic process.

In summary, the ALA-PTGS2 composite system exhibited high binding stability and strong hydrogen bonding interactions, suggesting positive ALA binding to PTGS2. PTGS2 is merely a predicted target rather than a definitively validated target of the extract. These molecular dynamics results are predictive and serve only as theoretical evidence.

### 3.10. CDDP-WE Combination Treatment Activates Tumor-Suppressive Pathways in CRC SW620 and HCT116 Cells

**Figure 9:**
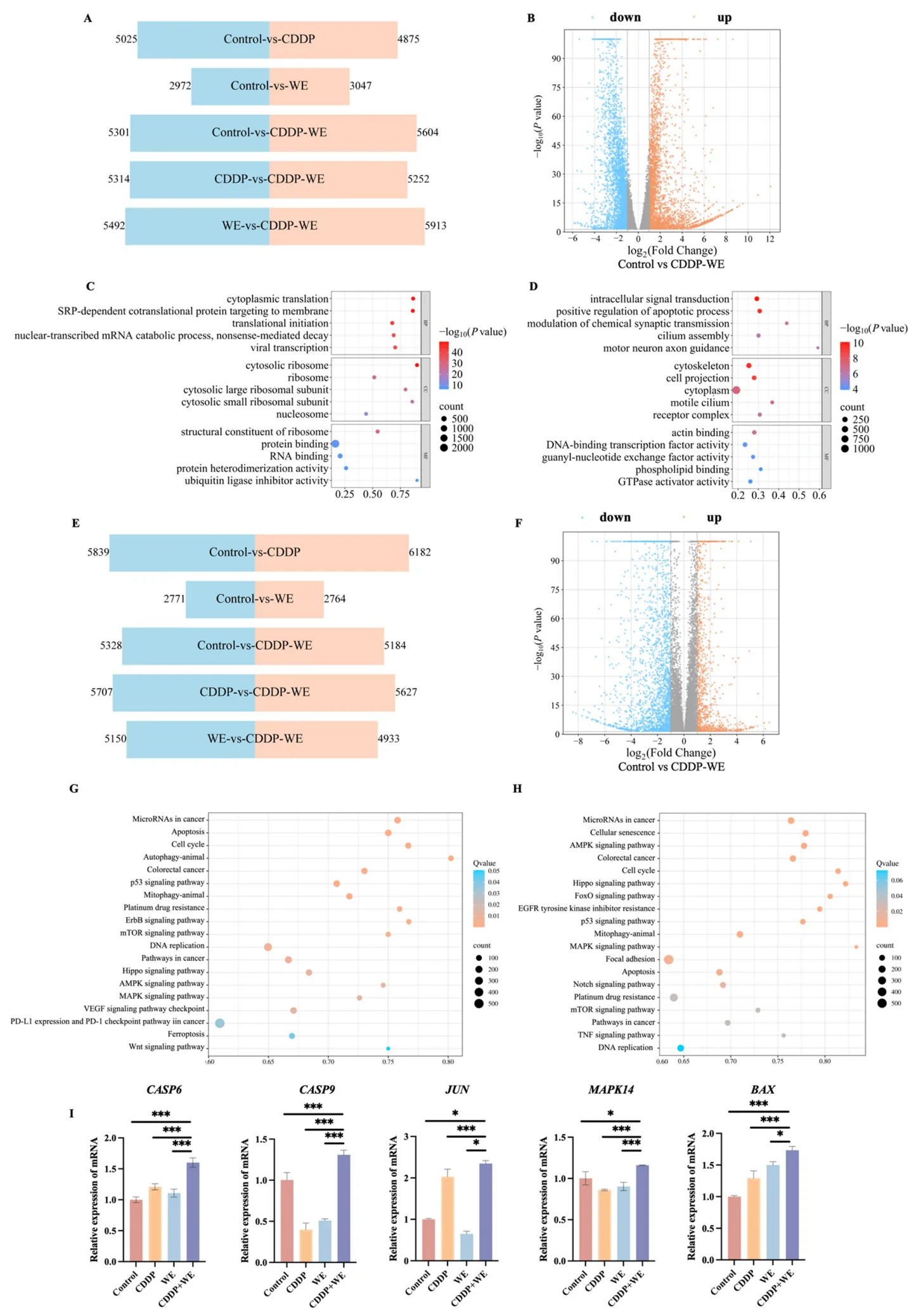
RNA sequencing (RNA-seq) analysis. (**A**,**E**) RNA-seq analysis showing numbers of differentially expressed genes in the indicated treatment comparisons for both SW620 and HCT116 cell lines. (**B**,**F**) Volcano plots of DEGs between control and CDDP-WE groups (**C**,**D**) GO enrichment analysis of the DEGs in the control vs. CDDP-WE group. (**G**,**H**) KEGG pathway enrichment analysis of the DEGs in the control vs. CDDP-WE group. (**I**) qPCR analysis of the mRNA expression levels of selected genes in SW620. The mean ± SD is presented, with *n* = 3. * *p* < 0.05, and *** *p* < 0.001 versus the indicated comparison groups.

To further investigate the molecular mechanisms underlying the CDDP-WE combination treatment-induced CRC cell death, transcriptome analysis was performed (data accession ID: PRJNA1492816 and SUB16317975). In the control and CDDP-WE groups, comparative transcriptomics identified 5301 downregulated and 5604 upregulated genes in SW620 cells, and 5328 downregulated and 5184 upregulated transcripts in HCT116 cells (*p* < 0.05) (Figure 9A,E). A cross-comparison revealed that 463 genes were upregulated and 739 were downregulated in both SW620 and HCT116 cells (fold change > 2 for upregulation and < 0.5 for downregulation, *p* < 0.05) (Figure 9B,F). Subsequent GO functional enrichment analysis (Figure 9C,D) and KEGG pathway enrichment analysis (Figure 9G,H) of the DEGs showed that pathways including apoptosis, p53 signaling, MAPK signaling, mTOR signaling, AMPK signaling, and mitophagy were enriched in both SW620 and HCT116 cells, suggesting that they may serve as key pathways in the anti-CRC effect exerted by the combination therapy. The expression of key genes (*CASP6*, *CASP9*, *MAPK14*, *BAX*, and *JUN*) related to apoptosis and oxidative stress injury was assessed via qRT-PCR. Compared to the control group or single-factor treatment, the mRNA expression levels of *CASP6*, *CASP9*, *MAPK14*, *BAX* and *JUN* in the CDDP-WE treatment were significantly upregulated in SW620. Collectively, our findings suggest that the WE may aggravate the effect of CDDP by affecting cell cycle- and oxidative stress-related pathways such as the p53 and MAPK signaling pathways (Figure 9I).

### 3.11. DIA-Based Proteomic Profiling Reveals the Impact of CDDP-WE Combination Therapy

**Figure 10:**
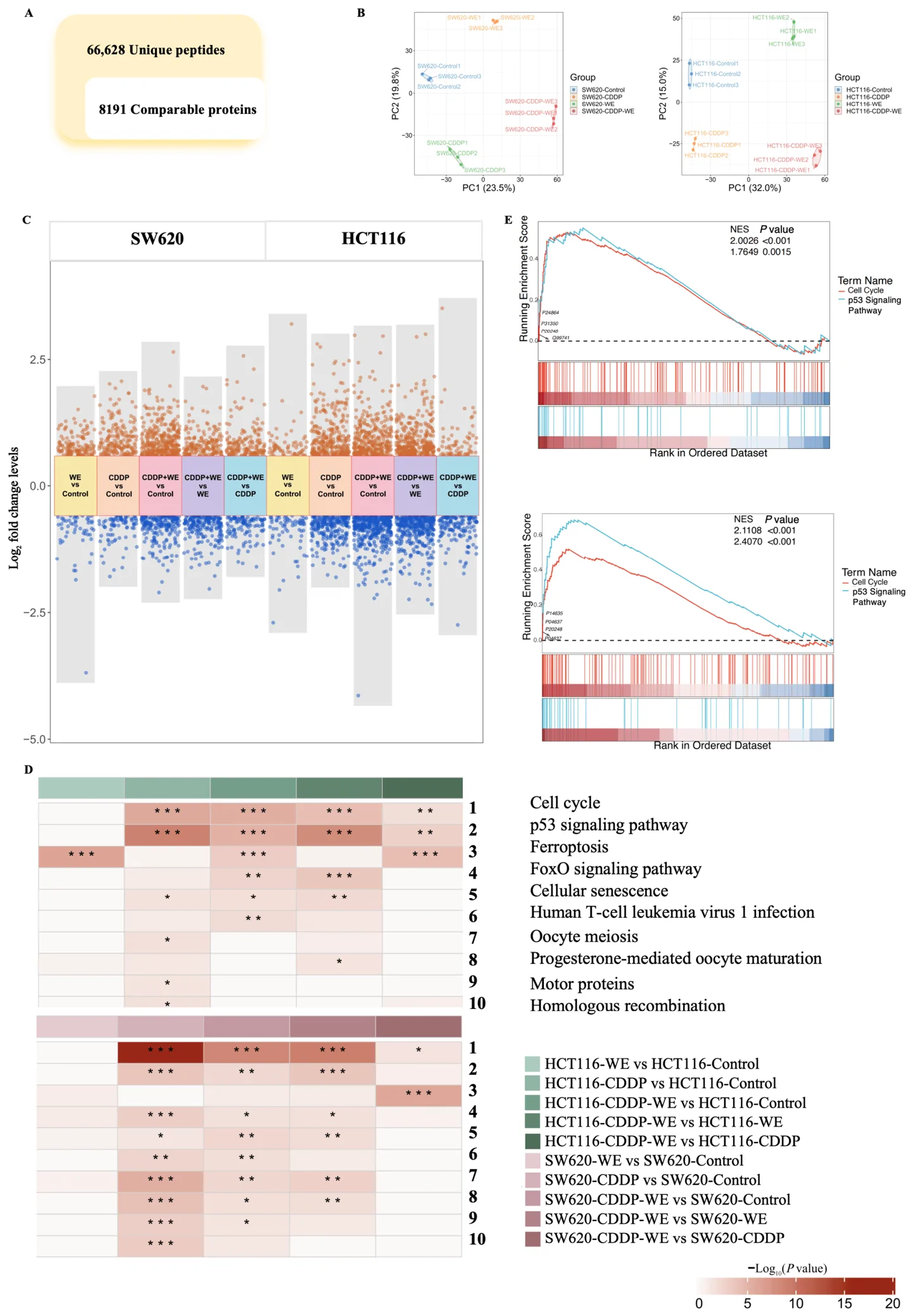
Quantitative proteomics in the SW620 and HCT116 cells treated with WE, CDDP, or combination therapy. (**A**) Quantitative proteome profiling identified 66,628 unique peptides, corresponding to 8191 comparable proteins. (**B**) Principal component analysis presentation. (**C**) Volcano plot of differential protein expression. (**D**) KEGG pathway enrichment analysis of differentially expressed proteins (DEPs). (**E**) GSEA analysis of the pathways as highlighted. In (**C**), orange and blue denote upregulated and downregulated proteins, respectively; grey bands separate the indicated comparisons. In (**D**), the color scale represents −log_10_(*p*); * *p* < 0.05, ** *p* < 0.01, and *** *p* < 0.001.

To further validate the underlying mechanisms of the anti-CRC synergistic effects of the CDDP-WE combination treatment, we investigated the impact of the treatment on the global protein expression through DIA-based proteomics analysis. In total, 66,628 peptides corresponding to 8191 proteins were identified at a 1% false discovery rate (FDR) in SW620 and HCT116 cells (Figure 10A) (data accession ID: PXD081174). PCA demonstrated the high reproducibility of the treatments (Figure 10B). Stringent thresholds were applied in quantitative proteomics (|log_2_FC| > 1.5). In the groups WE vs. control, CDDP vs. control, CDDP-WE vs. control, CDDP-WE vs. WE, and CDDP-WE vs. CDDP, we identified 133, 533, 721, 676, and 208 DEPs in HCT116 cells and 192, 261, 540, 363, and 269 DEPs in SW620 cells, respectively (Figure 10C). Notably, more DEPs were observed for the CDDP-WE treatment than for the individual CDDP and WE alone treatments, suggesting that the combination of CDDP-WE exerts an enhanced effect on CRC cells. Clusters of Orthologous Groups (COG) analysis revealed the top two pathways to be “general function prediction only” (16.60% in SW620 and 16.05% in HCT116) and “signal transduction mechanisms” (15.53% in SW620 and 15.89% in HCT116) (Figure S7A).

To further explore the proteomic changes related to the CDDP-WE treatment, DEPs were analyzed through GO, KEGG, and GSEA pathway enrichment analyses (Figure 10D,E and Figure S7B). GO enrichment (CDDP-WE vs. control) highlighted biological processes related to regulation of the cell cycle and DNA replication. KEGG enrichment analysis confirmed the involvement of oxidative stress and other mitochondrial-related pathways such as cell cycle, p53, ferroptosis and cellular senescence pathways.

### 3.12. Joint Analysis of Transcriptomes and Proteomes Reveals That CDDP-WE Treatment Has a Stronger Effect on Protein Functions

**Figure 11:**
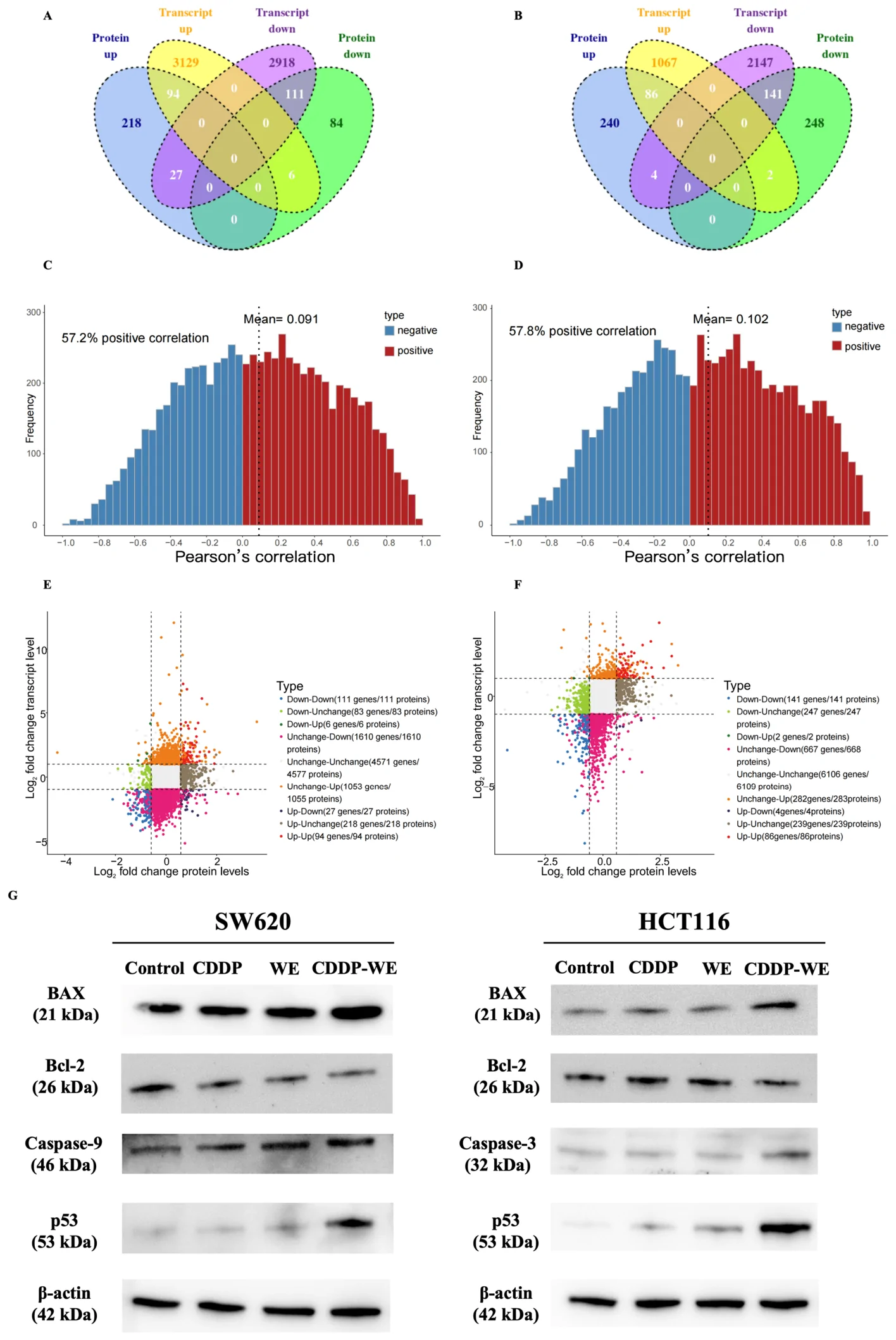
(**A**,**B**) Venn diagram illustrating the overlap of differentially expressed genes at transcript and protein levels. (**C**,**D**) Distribution of Pearson correlation coefficients between mRNA and protein expression. (**E**,**F**) Nine-quadrant plot for integrated transcriptomic–proteomic analysis. (**G**) Western blot of BAX, Bcl-2 and Caspase-9 (SW620), Caspase-3 (HCT116), and p53 in SW620 and HCT116 cells treated with or without CDDP alone, WE alone or the CDDP-WE combination. β-actin: Loading control. In (**A**,**B**), colors identify the labeled transcript and protein sets. In (**C**,**D**), red and blue indicate positive and negative correlations, respectively. In (**E**,**F**), colors identify the nine transcript–protein expression categories listed in the legends.

To explore the specific molecular mechanisms underlying the therapeutic effect of the CDDP-WE treatment on CRC, we performed integrated transcriptomic and proteomic correlation analyses to evaluate the concordance of gene expression regulation at the transcriptional and post-transcriptional levels. Venn diagram analysis was conducted to quantify the overlapping and specific differentially expressed molecules at the transcript and protein levels (Figure 11A,B). In the SW620 cell line, 94 co-upregulated genes and 111 co-downregulated genes were identified at both the transcriptomic and proteomic levels. In the HCT116 cell line, after the CDDP-WE intervention, 86 genes were consistently upregulated and 141 genes were consistently downregulated in both the transcriptome and proteome. Most differentially expressed genes and proteins exhibited expression changes specific to a single omics layer.

Pearson correlation analysis revealed a weak global correlation between mRNA and protein expression in both cell groups (Figure 11C,D), with mean correlation coefficients of 0.091 and 0.102, as well as 57.2% and 57.8% positive correlation pairs, in SW620 and HCT116, indicating that protein abundance was largely modulated by post-transcriptional rather than transcriptional regulation after the CDDP-WE treatment. The nine-quadrant diagram (Figure 11E,F) indicates that CDDP-WE can lead to changes in the expression of proteins and RNAs across nine quadrants in SW620 and HCT116 cells.

Western blot analysis was performed to detect the expression levels of apoptosis-related proteins in SW620 and HCT116 cancer cell lines. Notably, compared with the control and individual treatment groups, the protein levels of p53 and BAX notably increased after CDDP-WE treatment, while the abundance of anti-apoptotic Bcl-2 decreased. The CDDP-WE treatment exhibited a superior regulatory effect on these apoptotic proteins, with more significant upregulation of pro-apoptotic proteins and stronger suppression of Bcl-2 expression in both cell lines. Notably, the two cell lines exhibited distinct caspase activation patterns. HCT116 cells showed significantly upregulated Caspase-3 expression, while SW620 cells displayed increased Caspase-9 levels, indicating that the treatment induced cell apoptosis through different caspase-dependent pathways in these two colorectal cancer cell lines. These results demonstrate that the combination of CDDP and the WE exerted a synergistic pro-apoptotic effect on cancer cells via the p53-mediated apoptotic signaling pathway (Figure 11G).

### 3.13. The WE Contains a Number of Bioactive Ingredients That Enhance the Anticancer Effect of CDDP

**Figure 12:**
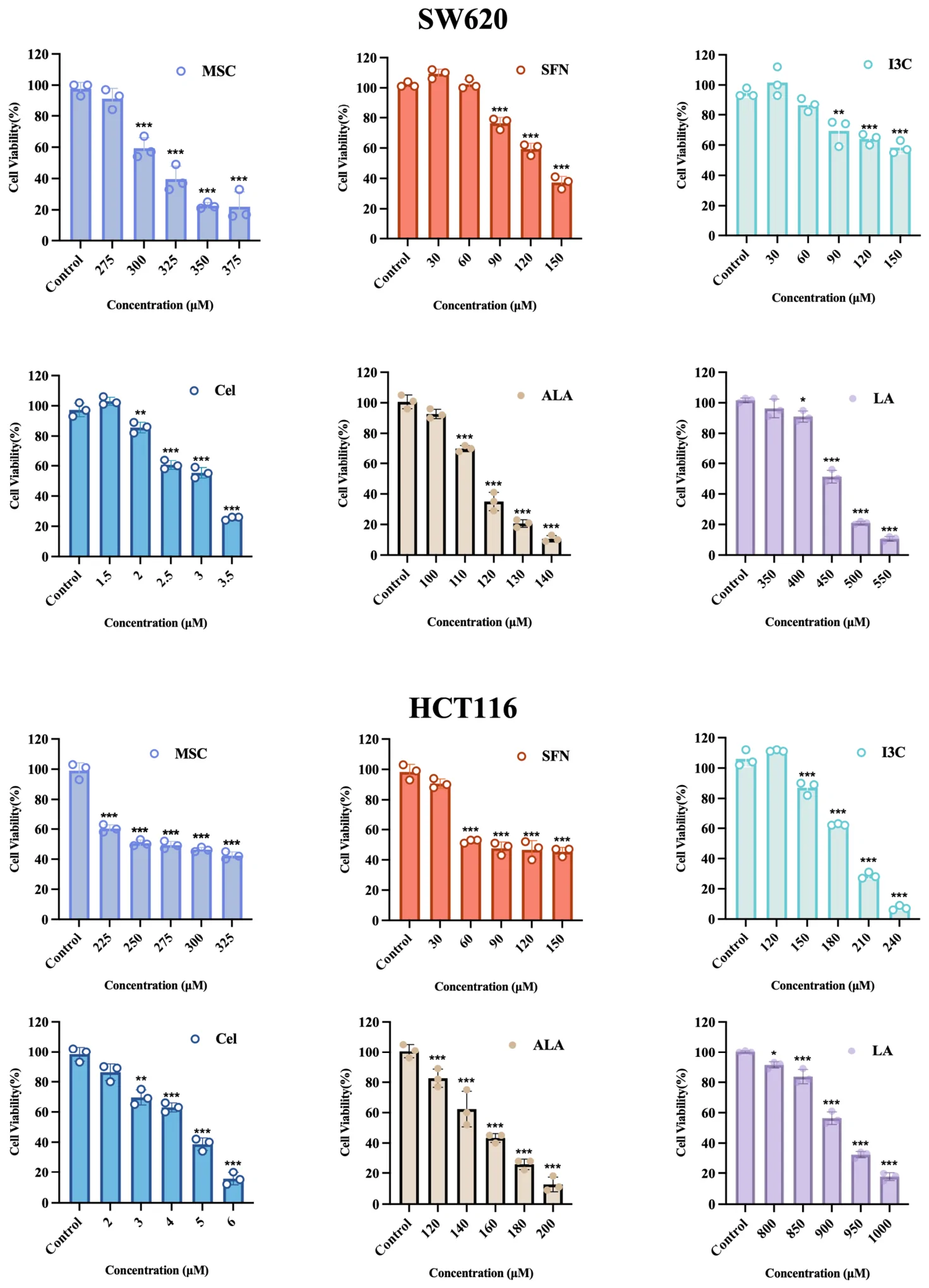
Effects of MSC, SFN, I3C, Cel, ALA and LA on SW620 and HCT116 cell viability. * *p* < 0.05, ** *p* < 0.01, and *** *p* < 0.001 vs. control group.

**Figure 13:**
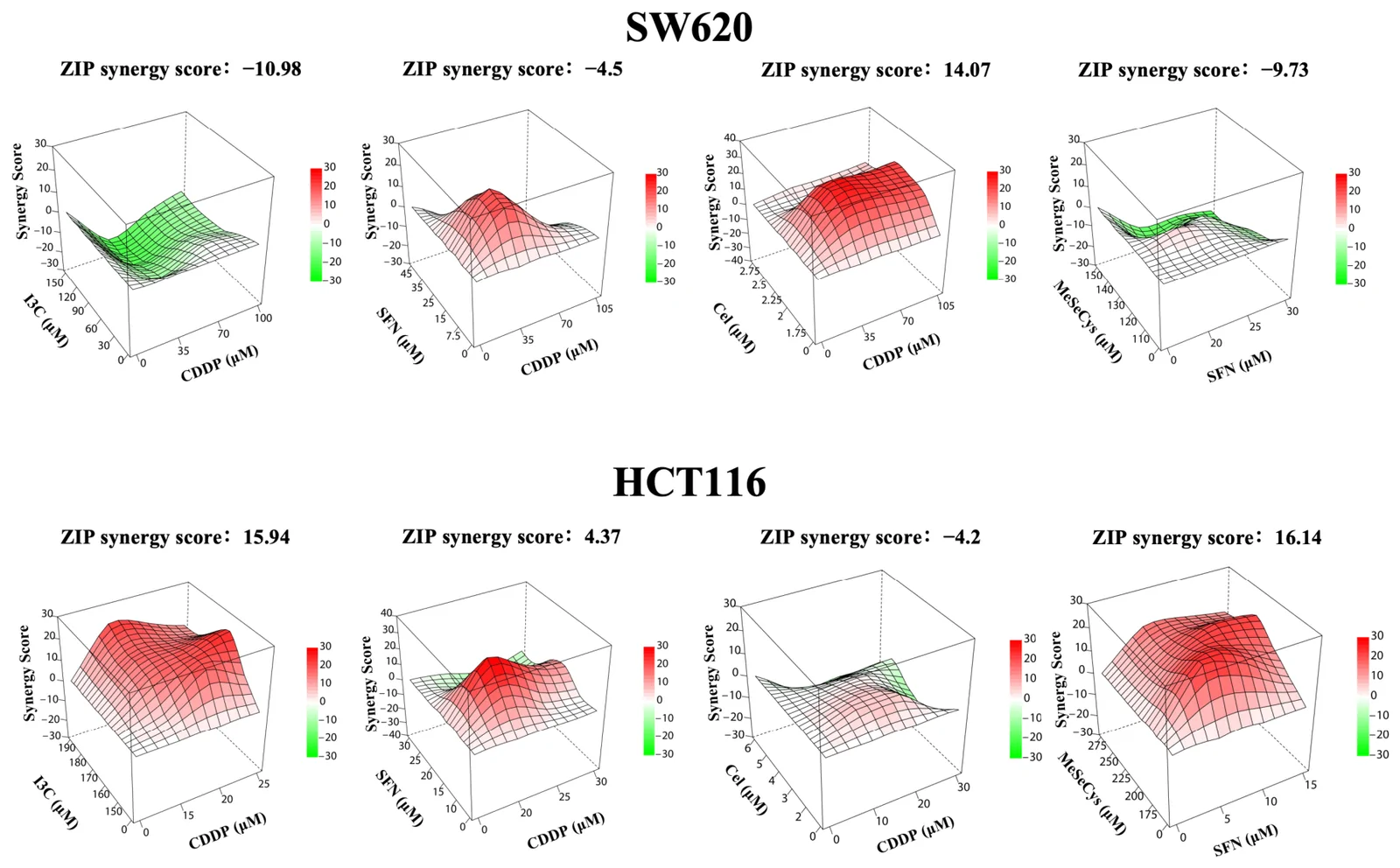
The synergy of the above active ingredients contained in the selenium-enriched rapeseeds.

Cruciferous plants have been considered to be functional foods for a long time. The beneficial anticancer effects of cruciferous vegetables are generally associated with glucosinolates and their degradation products, resveratrol and its derivatives, polyunsaturated fatty acids, vitamins, and minerals, which have broad potential applications in disease prevention and treatment. Studies have shown that ALA, as a promising dietary adjuvant, can enhance the efficacy of chemotherapy for colorectal cancer [36]. The potential of MSC combined with gemcitabine (GEM) chemotherapy has also been confirmed [37]. It is well known that SFN and I3C have been shown to have health-promoting effects [38]. Cel has shown active pharmacological activities in a variety of cancers, and clinical studies have shown its potential anticancer effect [39]. LA coordinates the dual antitumor response by promoting cancer cell apoptosis and regulating the tumor microenvironment [40]. To identify active ingredients in the WE with potential anti-CRC effects, we evaluated six prototype compounds (MSC, SFN, I3C, Cel, ALA, and LA) in SW620 and HCT116 CRC cell lines. SW620 and HCT116 cell viability assays revealed that all of the candidate active ingredients exhibited significant cytotoxicity (Figure 12). A synergistic effect was observed between CDDP and MSC in SW620 and HCT116 cells. In addition, combinations such as MSC-SFN (ZIP: 16.14), CDDP-I3C (ZIP: 15.94), and CDDP-SFN (ZIP: 4.37) exhibited synergistic inhibition only in HCT116 cells, while CDDP-Cel (ZIP: 14.07) exhibited synergistic inhibition only in SW620 cells (Figure 13). This study confirms the synergism between CDDP and other anticancer active ingredients in selenium-enriched rapeseed. These findings suggest that MSC, SFN, I3C, Cel, ALA, and LA are the candidate constituents in the WE responsible for arresting the cell cycle and promoting cell death.

### 3.14. WE Acute Toxicity Assay in Rats

**Figure 14:**
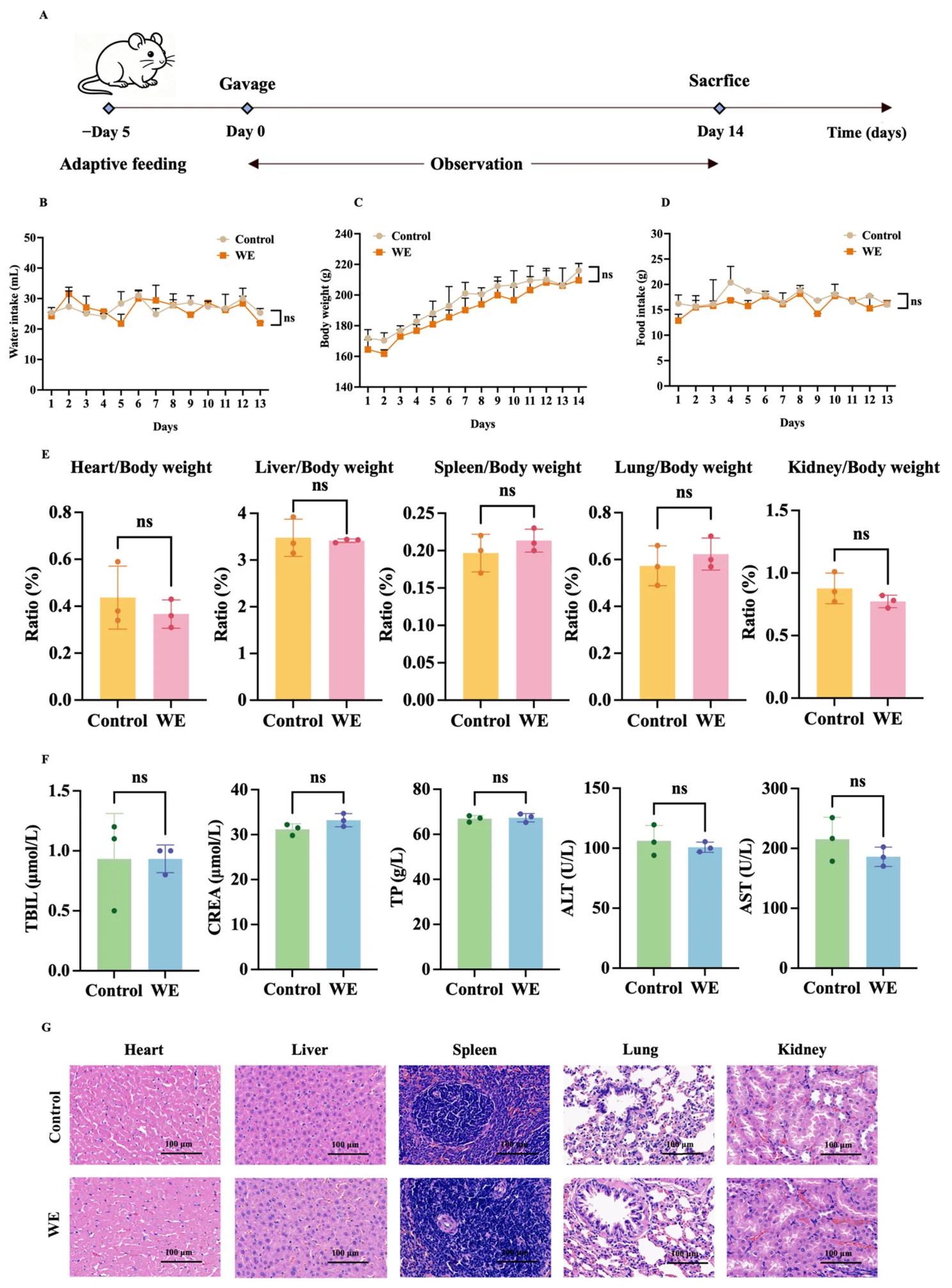
Acute toxicity assessment in rats following oral gavage of WE. (**A**) Schematic illustration of the acute toxicity experimental schedule. (**B**) Daily water intake, (**C**) body weight, and (**D**) daily food consumption of rats throughout observation. (**E**) Organ coefficients of major organs. (**F**) Serum function biochemical indicators of rats. Data in (**B**–**F**) are shown as mean ± SD (*n* = 3). (**G**) Representative HE-stained micrographs of heart, liver, spleen, lung and kidney tissues for the control and WE group. Scale bar: 100 μm.

In order to investigate the biological safety of the WE, we selected female rats recommended by the OECD to evaluate the acute toxicity of the extract (Figure 14A). During the two-week observation period, appearance, behavior, and lifestyle habits were recorded, and no clear symptoms of intoxication in the Sprague Dawley (SD) rats were observed. Additionally, no treatment-related deaths occurred. Compared with the control group, there were no significant changes in food intake, water intake, and weight gain in the WE group (Figure 14B–D). Therefore, the LD50 value of the WE exceeded 2000 mg/kg.

The rats were sacrificed after the two-week experimental period, and organs/tissues (the heart, liver, spleen, lungs, and kidneys) were dissected. No significant difference was observed in the appearance (Figure S9) or the organ/tissue–body weight ratios of these organs (Figure 14E). In addition, evaluation through hematological analysis showed that the WBC, LYM, MONO, NEUT, EO, BASO, RBC, HGB, and PLT indices were comparable between the control and WE groups (Figure 14F; Table S3). Finally, histological analysis via HE staining did not reveal a notable difference in these internal organs between the two groups (Figure 14G). We concluded that the WE showed limited toxicity to the animals during the experimental period.

## 4. Discussion

In CRC treatment, traditional chemotherapy using drugs such as CDDP is initially effective but often leads to drug resistance and disease recurrence. One way to address this problem is to increase the drug dosage; however, this inevitably increases the risk of toxicity to normal cells. Therefore, the search for safe dietary natural products that can enhance or work synergistically with the chemotherapy drug has become the focus of extensive and ongoing research [41,42]. The wide variety of plant metabolites provides an ideal resource for identifying such natural products [43]. Notably, it is found that combining a mixture of natural products with chemotherapy drugs often yields more efficient outcomes. In this report, we present strong evidence showing that extracts obtained from the inflorescence of selenium-enriched oilseeds not only exerted anticancer function alone but also enhanced the anticancer function of CDDP.

The mechanism of CDDP mainly relies on mediating oxidative stress [44], inducing mitochondrial dysfunction [45], and forming Pt-DNA adducts by cross-linking with the DNA of CRC cells to inhibit DNA replication [46]. It also plays an important role by altering the composition of the cancer microenvironment (such as recruiting macrophages, antigen-presenting cells, myeloid cells and cancer-related fibroblasts) in vivo [47]. Previous studies have reported that lycopene enhances the sensitivity of oral squamous cell carcinoma to CDDP by inhibiting the PI3K/Akt signaling pathway and reversing epithelial–mesenchymal transition [48]. Our study found that the WE significantly enhanced the function of CDDP in inducing CRC cell apoptosis. In addition, the WE enhanced the function of CDDP in promoting the release of LDH, the depletion of GSH, and an increase in ROS, thereby triggering enhanced oxidative stress in CRC cells. Further studies showed that the WE enhanced the function of CDDP in triggering a decrease in mitochondrial membrane potential, thereby activating the intrinsic mitochondrial apoptosis pathway.

Natural products show the potential to reverse the cellular damage caused by chemotherapy drugs. For example, a Au-M nanosystem elicited potent renoprotection via Nrf2-orchestrated transcriptional reprogramming in vivo [49]. Icaritin (ICT) is an active flavonoid isolated from Epimedium plants, and studies have found that it can maintain the anticancer efficacy of CDDP while reducing its nephrotoxicity [8]. The natural food-derived ingredient corn silk extract (CSE) can reduce the levels of myocardial injury markers and provides a novel mechanistic basis for supporting cardiovascular health [50]. We found that the WE not only enhanced the sensitivity of CRC cells to CDDP but also had no cytotoxic effect on NCM460 and HK-2 after 24 h and 48 h of treatment (Figure S8). Using LC-MS/MS systems and HPLC-ICP-MS for analysis, we found that MSC was the main selenospecies in the WE. Multiple preclinical studies have demonstrated that selenium compounds can cooperate with CDDP to enhance antitumor activity and mitigate CDDP-induced organ toxicity [51]. Prior research has predominantly relied on purified selenium monomers or artificially synthesized selenium nanomaterials to achieve combinatorial effects with platinum drugs. Consistent with these findings, our results confirmed synergistic anti-CRC effects between selenium-enriched rapeseed extracts and CDDP. Follow-up cellular assays further revealed that MSC, I3C, Cel, ALA, LA, and SFN were the candidate bioactive components exerting significant inhibitory effects on CRC. These compounds enhanced the antitumor effects when combined with CDDP. Nevertheless, the phytochemical composition of rapeseed extracts is far more complex than that of individual selenium compounds, consisting of diverse selenium species, polyunsaturated fatty acids, and glucosinolate metabolites, which jointly mediate the synergistic responses observed in CRC cells. Overall, this study highlights the potential of selenium-enriched rapeseed extracts as natural chemosensitizers, laying a foundation for subsequent in vivo validation and translational exploration.

Key targets BCL2, BAX, p53, CASP3 and CASP9 modulate proliferation, apoptosis and CDDP resistance in CRC [52,53,54]. Anti-apoptotic BCL2 inhibits mitochondrial permeabilization, whereas pro-apoptotic BAX triggers mitochondrial injury [55]. p53 mediates DNA damage responses, cell cycle arrest and apoptosis [56]. CASP3 is the executioner caspase and CASP9 is the initiator caspase under mitochondrial stress. These hubs connect MAPK and apoptotic pathways: MAPK regulates p53 activity to tune BAX/BCL2 balance and cell fate [57]. Together, these nodes integrate cell cycle, MAPK and apoptotic cascades to govern CRC proliferation, apoptosis and CDDP sensitivity. In this study, it was demonstrated that WE modulated the expression of the proteins Bcl-2, BAX, Caspase-3, Caspase-9 and p53, ultimately enhancing CDDP-induced apoptosis in CRC cells.

In conclusion, our studies provide evidence showing that selenium-enriched rapeseed may be a useful source for identifying natural products that can be used against CRC cells. Although the CDDP-WE combination shows promising results, its practical application still faces several challenges. Considering the long-term safety of the extracts, it is important to optimize the dose and treatment strategy, and systematic evaluation is needed. The current cell model may not fully reflect the complexity of the human cancer microenvironment, and the potential side effects also need to be further explored in animal experiments. In addition, the immunomodulatory effect of the active ingredient combination and its joint administration with existing cancer treatments (such as chemotherapy, radiotherapy, and targeted therapy) are worthy of further study. Clinical trials are crucial for translating these findings into practice. Our future research will focus on these limitations.

## Data Availability

The data presented in this study are openly available in the National Center for Biotechnology Information (NCBI) with accession number [PRJNA1492816]. Raw proteomic data are available in iProX (PXD081174, IPX0018391000).

## Supplementary Materials

The following supporting information can be downloaded at: https://www.mdpi.com/article/10.3390/cells15211934/s1, Figure S1. The effects of methanol concentration on the viability of cells treated with CE alone and co-treated with CE and CDDP. Figure S2: The effects of CDDP and WE alone or in combination on the viability of CRC cells. Figure S3: Determination of ABTS free radical scavenging activity of WE. Figure S4: Total ion chromatogram of WE in positive ion mode and negative ion mode. Figure S5: Molecular dynamics simulation. Figure S6: Systematic analyses of the ALA-PTGS2 complex. Figure S7: Proteomic related analysis. Figure S8: Cell viability of NCM460 cells treated by WE. Figure S9. Representative gross appearance of rat major organs after acute toxicity treatment. Table S1: HPLC-ICP-MS results for selenium speciation. Table S2: Content of total and individual fatty acid species. Table S3. Hematological parameters of rats after different treatments. Table S4. Primer sequences for qRT-PCR. Table S5. Binding free energy of active ingredients with proteins by molecular docking.

## Abbreviations

The following abbreviations are used in this manuscript:

ABTS	2,2′-azino-bis (3-ethylbenzothiazoline-6-sulfonic acid)
ALA	α-linolenic acid
ALT	Alanine aminotransferase
ANOVA	One-way analysis of variance
AST	Aspartate aminotransferase
BE	n-butanol extract
CCK-8	Cell Counting Kit-8
CDDP	Cisplatin
CDDP-WE	Cisplatin and water extract
cDNA	Complementary DNA
CE	Crude extract
CEs	Crude extracts
Cel	Celastrol
COG	Clusters of Orthologous Groups
CRC	Colorectal cancer
CREA	Creatinine
CSE	Corn silk extract
DEGs	Differentially expressed genes
DEPs	Differentially expressed proteins
EAE	Ethyl acetate extract
EdU	ethynyl-2′-deoxyuridine cell proliferation kit
EPA	Eicosapentaenoic acid
FAs	Fatty acids
FC	Fold change
FDA	Food and Drug Administration
FDR	False discovery rate
FEL	Free energy landscape
FITC	Fluorescein isothiocyanate
GO	Gene ontology
GPs	Glycerophospholipids
GSH	Reduced glutathione assay kit
HE	Hematoxylin and eosin
HPLC-ICP-MS	High-performance liquid chromatography–inductively coupled plasma–mass spectrometry
I3C	Indole-3-carbinol
ICT	Icaritin
IC50	Half-maximal inhibitory concentration
KEGG	Kyoto encyclopedia of genes and genomes
LA	Linoleic acid
LC-MS/MS	Liquid chromatography-tandem mass spectrometry
L-LDH	L-lactate dehydrogenase
MSC	Methylselenocysteine
MMP	Mitochondrial membrane potential
MMP3	Matrix metalloproteinase 3
MM/PBSA	Molecular mechanics/Poisson–Boltzmann surface area
Na_2_SeO_3_	Sodium sulfite
Na_2_SeO_4_	Sodium selenate
OECD	Organization for Economic Cooperation and Development
PEE	Petroleum ether extract
PI	Propidium iodide
PME	Particle mesh Ewald
Rg	Radius of gyration
RIPA	Radio-Immunoprecipitation Assay
RMSD	Root mean square deviation
RMSF	Root mean square fluctuation
RNA-seq	RNA sequencing
ROS	Reactive oxygen species
Resolvin D5	RvD5
SASA	Solvent-accessible surface area
SD	Standard deviation
SE	Selenium-enriched rapeseed starting material
SeCys_2_	Selenocysteine
SeIV	Selenite
SeMet	Selenomethionine
SeVI	Selenate
SFN	Sulforaphane
TBIL	Total bilirubin
TEM	Transmission electron microscopy
TP	Total protein
TUNEL	TdT-mediated dUTP Nick-end labeling
UPLC-MS/MS	Ultra-performance liquid chromatography-tandem mass spectrometry
WE	Water extract
ZJU	Zhejiang University

## References

[1] Eng C., Yoshino T., Ruíz-García E., Mostafa N., Cann C.G., O’Brian B., Benny A., Perez R.O., Cremolini C. (2024). Colorectal Cancer. Lancet.

[2] Siegel R.L., Wagle N.S., Cercek A., Smith R.A., Jemal A. (2023). Colorectal Cancer Statistics, 2023. CA A Cancer J. Clin..

[3] Kuboki Y., Fakih M., Strickler J., Yaeger R., Masuishi T., Kim E.J., Bestvina C.M., Kopetz S., Falchook G.S., Langer C. (2024). Sotorasib with Panitumumab in Chemotherapy-Refractory KRASG12C-Mutated Colorectal Cancer: A Phase 1b Trial. Nat. Med..

[4] Trefny M.P., Kroemer G., Zitvogel L., Kobold S. (2025). Metabolites as Agents and Targets for Cancer Immunotherapy. Nat. Rev. Drug Discov..

[5] Singh M., Morris V.K., Bandey I.N., Hong D.S., Kopetz S. (2024). Advancements in Combining Targeted Therapy and Immunotherapy for Colorectal Cancer. Trends Cancer.

[6] Yu L., Yuan J., Lou S., Huang A., Cai X., Ji J., Dai X., Mao X., Mao Y., Sun L. (2025). Factors Affecting Survival in Patients with Colorectal Cancer: An Umbrella Review. J. Transl. Med..

[7] Garbo S., Di Giacomo S., Łażewska D., Honkisz-Orzechowska E., Di Sotto A., Fioravanti R., Zwergel C., Battistelli C. (2022). Selenium-Containing Agents Acting on Cancer—A New Hope?. Pharmaceutics.

[8] Luo P., Chen J., An Y., Meng K., Zhou W., Li W., Liu J., Zhao W., He W., Cao T. (2026). Icaritin Ameliorates Cisplatin-Induced Mitochondrial Metabolic Dysfunction-Associated Nephrotoxicity and Synergistically Potentiates Its Antitumor Efficacy. Adv. Sci..

[9] González Sánchez S., García Fernández J., Cascales-Campos P.A., Gonzalez Gil A., Manzanedo I., Pereira Perez F., Díaz Gómez D., González-de Pedro C., Asensio Diaz E., Pacheco Sanchez D. (2025). Interval Cytoreductive Surgery and Cisplatin- or Paclitaxel-Based HIPEC for Advanced Ovarian Cancer. JAMA Netw. Open.

[10] Zhang X., Ning S., Fang F., Zhou C., Yang Z., Cao L., Wang K., Feng Z., Li B., Luo D. (2026). Leveraging Cisplatin-Derived Prodrugs as Helper Lipids in LNPs to Boost the Efficacy of Cancer Chemoimmunotherapy. ACS Nano.

[11] Elmorsy E.A., Saber S., Hamad R.S., Abdel-Reheim M.A., El-kott A.F., AlShehri M.A., Morsy K., Salama S.A., Youssef M.E. (2024). Advances in Understanding Cisplatin-Induced Toxicity: Molecular Mechanisms and Protective Strategies. Eur. J. Pharm. Sci..

[12] Zhang M., Sun T., Wang X., Zhang Z., Zhai S., Hou X., Hou Y., Bai G. (2025). Structure Characterization of a Polysaccharide Isolated from Sugarcane Leaves and Its Macrophage Polarization Activity and Beneficial Effects for Cisplatin Treatment of Lung Cancer. Carbohydr. Polym..

[13] Hamza A.A., Heeba G.H., Hassanin S.O., Elwy H.M., Bekhit A.A., Amin A. (2023). Hibiscus-Cisplatin Combination Treatment Decreases Liver Toxicity in Rats While Increasing Toxicity in Lung Cancer Cells via Oxidative Stress- Apoptosis Pathway. Biomed. Pharmacother..

[14] Zhang Y., Lv X., Wang D., Zheng C., Chen H., Yuan Y., Wei F. (2025). Metabolomics Combined with Biochemical Analyses Revealed Phenolic Profiles and Antioxidant Properties of Rapeseeds. Food Chem..

[15] Salami M., Heidari B., Batley J., Wang J., Tan X.-L., Richards C., Tan H. (2024). Integration of Genome-Wide Association Studies, Metabolomics, and Transcriptomics Reveals Phenolic Acid- and Flavonoid-Associated Genes and Their Regulatory Elements under Drought Stress in Rapeseed Flowers. Front. Plant Sci..

[16] Su C.-C., Yu C.-C., Shih Y.-W., Liu K.-L., Chen H.-W., Wu C.-C., Yang Y.-C., Yeh E.-L., Li C.-C. (2023). Protective Effect of Alpha-Linolenic Acid on Human Oral Squamous Cell Carcinoma Metastasis and Apoptotic Cell Death. Nutrients.

[17] Kim J.S., Han S., Kim H., Won S.Y., Park H.W., Choi H., Choi M., Lee M.Y., Ha I.J., Lee S.-G. (2022). Anticancer Effects of High Glucosinolate Synthesis Lines of Brassica Rapa on Colorectal Cancer Cells. Antioxidants.

[18] Lu Y., Fang X., Dun X., Wang X., Jiang L., Wang H., Chen Z. (2025). Integrated Multi-Omics Analysis Reveals the Bioactive Components and Mechanism of Selenium-Enriched Rapeseed in Enhancing Male Reproductive Function. Food Biosci..

[19] Che H., Lu Y., Li T., Fang X., Wang X., Wang H., Dun X., Chen Z. (2025). Investigation into Bioactive Selenium Species and the Mechanism of Action Behind Selenium-Enriched Rapeseed Flowering Stalks Alleviating Cadmium-Induced Toxicity in Mouse Sertoli Cells. Antioxidants.

[20] Lin Y., Wang T., Wu Z., Wang L., Yu Z., Wang J., Liu T., Cai X., Li H., Ren Y. (2026). Therapeutic Effects of Selenium-Enriched Rapeseed against Triple-Negative Breast Cancer: Involvement of Resolvin D5 Activation and IL-17 Signaling Inhibition. Food Funct..

[21] Zhang Z., Liu Y., Feng W., Mao P., Yang J., Zhao Z., Zhou S., Zhao A.Z., Li F., Mu Y. (2024). Omega-3 Polyunsaturated Fatty Acids Protect against Cisplatin-Induced Nephrotoxicity by Activating the Nrf2 Signaling Pathway. Int. J. Biol. Macromol..

[22] Feng S., Xie X., Chen C., Zuo S., Zhao X., Li H. (2022). Alpha-Linolenic Acid Inhibits Hepatocellular Carcinoma Cell Growth through Farnesoid X Receptor/β-Catenin Signaling Pathway. Nutr. Metab..

[23] Zhan J.-P., Qi M., Wang C., Wang X.-F., Wang H.-Z., Dun X.-L. (2024). Precise Determination of Selenium Forms and Contents in Selenium-Enriched Rapeseed Seedlings and Flowering Stalks by HPLC-ICP-MS. J. Agric. Food Chem..

[24] Whiting F.J.H., Mossner M., Gabbutt C., Kimberley C., Barnes C.P., Baker A.-M., Yara-Romero E., Sottoriva A., Nichols R.A., Graham T.A. (2025). Quantitative Measurement of Phenotype Dynamics during Cancer Drug Resistance Evolution Using Genetic Barcoding. Nat. Commun..

[25] Wu M., Liu H., Shen J., Xie Z., Yang S., Guo J., Liu Y., Lian H., Wang D. (2025). Utilizing Allelopathy from the Invasive Plant Solidago Canadensis to Control Microcystis Aeruginosa Blooms: An Integrated Metabolomic and Bioassay Approach. J. Hazard. Mater..

[26] Zhu Y., Cao Z., Xu F., Huang Y., Chen M., Guo W., Zhou W., Zhu J., Meng J., Zou J. (2012). Analysis of Gene Expression Profiles of Two Near-Isogenic Lines Differing at a QTL Region Affecting Oil Content at High Temperatures during Seed Maturation in Oilseed Rape (*Brassica napus* L.). Theor. Appl. Genet..

[27] Velankar S., Burley S.K., Kurisu G., Hoch J.C., Markley J.L., Owens R.J. (2021). The Protein Data Bank Archive. Structural Proteomics.

[28] Makarewicz T., Kaźmierkiewicz R. (2013). Molecular Dynamics Simulation by GROMACS Using GUI Plugin for PyMOL. J. Chem. Inf. Model..

[29] Morris G.M., Huey R., Lindstrom W., Sanner M.F., Belew R.K., Goodsell D.S., Olson A.J. (2009). AutoDock4 and AutoDockTools4: Automated Docking with Selective Receptor Flexibility. J. Comput. Chem..

[30] Trott O., Olson A.J. (2010). AutoDock Vina: Improving the Speed and Accuracy of Docking with a New Scoring Function, Efficient Optimization, and Multithreading. J. Comput. Chem..

[31] Páll S., Zhmurov A., Bauer P., Abraham M., Lundborg M., Gray A., Hess B., Lindahl E. (2020). Heterogeneous Parallelization and Acceleration of Molecular Dynamics Simulations in GROMACS. J. Chem. Phys..

[32] Wang J., Wolf R.M., Caldwell J.W., Kollman P.A., Case D.A. (2004). Development and Testing of a General Amber Force Field. J. Comput. Chem..

[33] Maier J.A., Martinez C., Kasavajhala K., Wickstrom L., Hauser K.E., Simmerling C. (2015). ff14SB: Improving the Accuracy of Protein Side Chain and Backbone Parameters from ff99SB. J. Chem. Theory Comput..

[34] Li F., Zhang X.-Q., Ho W., Tang M., Li Z., Bu L., Xu X. (2023). mRNA Lipid Nanoparticle-Mediated Pyroptosis Sensitizes Immunologically Cold Tumors to Checkpoint Immunotherapy. Nat. Commun..

[35] Adtani P.N., Al-Bayati S.A.A.F., Elsayed W.S. (2025). Sulforaphane from Brassica Oleracea Induces Apoptosis in Oral Squamous Carcinoma Cells via P53 Activation and Mitochondrial Membrane Potential Dysfunction. Pharmaceuticals.

[36] Li S., Lv T., Fan X., Feng M., Zhou M., Li Z. (2025). Alpha-Linolenic Acid Reverses Colorectal Cancer Drug Resistance by Suppressing Secreted Phosphoprotein 1 Expression and Tumor Stemness. Phytomedicine.

[37] Behera C., Sandha K.K., Banjare N., Malik S.B., Tabassum M., Kumar R., Kumar A., Mondhe D.M., Gupta P.N. (2022). Implication of Methylselenocysteine in Combination Chemotherapy with Gemcitabine for Improved Anticancer Efficacy. Eur. J. Pharm. Sci..

[38] Syed R.U., Moni S.S., Break M.K.B., Khojali W.M.A., Jafar M., Alshammari M.D., Abdelsalam K., Taymour S., Alreshidi K.S.M., Elhassan Taha M.M. (2023). Broccoli: A Multi-Faceted Vegetable for Health: An In-Depth Review of Its Nutritional Attributes, Antimicrobial Abilities, and Anti-Inflammatory Properties. Antibiotics.

[39] Zhang H., Huang J., Li Y., Jin W., Wei J., Ma N., Shen L., Gu M., Mu C., Xu D. (2025). Celastrol-Loaded Ginsenoside Rg3 Liposomes Boost Immunotherapy by Remodeling Obesity-Related Immunosuppressive Tumor Microenvironment in Melanoma. Acta Pharm. Sin. B.

[40] Yao Y., Xu C., Shao Y., Xu X., Yin Q., Cao X., Zheng M., Zhao D., Zheng W., Ni J. (2025). Linoleic Acid Potentiates Response to Chemotherapy in Biliary Tract Cancer Through RARγ Activation. FASEB J..

[41] Zhao J.Z., Li Y.F., Yuan F.K., Zhao M.L., Han Y.W., Wang J.X., Yang Q., Ye H.Y., Lu Y.C., Lee S.C. (2025). Okanin Suppresses the Growth of Colorectal Cancer Cells by Targeting at Peroxiredoxin 5. Adv. Sci..

[42] Lee J.H., Seo S.H., Shim J., Kim Y.-N., Yoon K. (2025). Narciclasine Enhances Cisplatin-Induced Apoptotic Cell Death by Inducing Unfolded Protein Response-Mediated Regulation of NOXA and MCL1. Cell. Mol. Biol. Lett..

[43] Berman T.A., Ben-Arye E., Kienle G.S., Master V., Huston A., Paller C.J. (2025). Integrating Botanicals into Oncology Care: Consideration of FDA Regulation of Botanical Products and Botanical Clinical Trials. Clin. Cancer Res..

[44] Soltau C.P., Tay B.Q., Richard D.J., Bottle S.E., Adams M.N. (2026). Steroidal-Nitroxide Hybrid Sustains Oxidative Stress and Enhances Cisplatin Efficacy in Vitro across Multiple Cancer Cell Lines. Biomed. Pharmacother..

[45] Huang Y., Tang D., Zhao R., Zhang J., Qu X., Li N., Ren Y., Luo X. (2026). Network Pharmacology Reveals That Yanghe Decoction Inhibits Osteosarcoma Progression via ROS-Induced Mitochondrial Dysfunction and Enhances Cisplatin Sensitivity. Genes Dis..

[46] Chen G., Li X., Huang Y., Zheng C., Fang H., Zhang Q., Liu K., Wu K., Zhu Q., Guo C. (2026). X-Ray Activated Platinum Complex Induces DNA Damage and Enhances Cancer Immunotherapy through Abscopal Effect. Nat. Biomed. Eng..

[47] Yang C., Deng X., Tang Y., Tang H., Xia C. (2024). Natural Products Reverse Cisplatin Resistance in the Hypoxic Tumor Microenvironment. Cancer Lett..

[48] Wang R., Lu X., Yu R., Shan Z. (2026). Lycopene Enhances the Sensitivity of Oral Squamous Cell Carcinoma to Cisplatin Through Inhibition of the PI3K/Akt Signaling Pathway and Reversal of Epithelial-Mesenchymal Transition. Drug Des. Dev. Ther..

[49] Fang C., Cai Y., He C., Lu Y., Wang X., Cai Y. (2026). Hydroxyl-Augmented Gold Nanorods via Lactone Ring-Opening Alleviate Cisplatin Nephrotoxicity through Nrf2 Activation. Biomaterials.

[50] Yang Q., Li Y., Qi L., Wang J., Liu M., Huang J., Sun M., Fu Y., Lin H., Lin Z. (2026). Corn Silk Extract Ameliorates Cisplatin-Induced Cardiac Injury by Restoring Mitochondrial Function and Suppressing Ferroptosis. Food Funct..

[51] Iglesias-Jiménez A., Artiaga G., Moreno-Gordaliza E., Milagros Gómez-Gómez M. (2025). Metallomic Evaluation of Selenium Nanoparticles and Selenomethionine for the Attenuation of Cisplatin-Induced Nephrotoxicity. Eur. J. Pharm. Biopharm..

[52] Ebrahimi M., Babaei E., Neri F., Feizi M.A.H. (2021). Anti-Proliferative and Apoptotic Effect of Gemini Curcumin in P53-Wild Type and P53-Mutant Colorectal Cancer Cell Lines. Int. J. Pharm..

[53] Kandhavelu J., Subramanian K., Naidoo V., Sebastianelli G., Doan P., Konda Mani S., Yapislar H., Haciosmanoglu E., Arslan L., Ozer S. (2024). A Novel EGFR Inhibitor, HNPMI, Regulates Apoptosis and Oncogenesis by Modulating BCL-2/BAX and P53 in Colon Cancer. Br. J. Pharmacol..

[54] Jayathilake A.G., Kadife E., Luwor R.B., Nurgali K., Su X.Q. (2019). Krill Oil Extract Suppresses the Proliferation of Colorectal Cancer Cells through Activation of Caspase 3/9. Nutr. Metab..

[55] Sahoo R.K., Tripathi S.K., Barik K., Biswal S., Patra S.K., Panda S.S., Kumar A., Biswal B.K. (2025). Piperlongumine Triggers Reactive Oxygen Species Driven Mitochondrial Dysfunction Leading to Apoptosis in Human Colon Cancer Cells. BioFactors.

[56] Suwanmanee G., Kheolamai P., Tantrawatpan C., Grimes D., Matei I.V., Paraoan L., Manochantr S. (2025). Fucoxanthin Protects Placenta-Derived Human Mesenchymal Stem Cells against Oxidative Stress-Induced Apoptosis by Modulating Genes Involved in DNA Damage Repair, ER Stress Response, P53-Induced Apoptosis. Stem Cell Res. Ther..

[57] Zhang Z., Li X., Zhang Y., Zhu H., Qiao Z., Lu Y., Mi X., Cao H., Shen G., He S. (2024). Absent in Melanoma 2 Attenuates Proliferation and Migration and Promotes Apoptosis of Human Colorectal Cancer Cells by Activating P38MAPK Signaling Pathway. Oncol. Res..

