# Supplementary material for: Selenium-Enriched Rapeseed Extract Synergizes with Chemotherapy Drug Cisplatin in Inhibiting Proliferation and Promoting Apoptosis of Colorectal Cancer Cells
Source: Cells. 2026 Oct 24;15(21):1934. doi: 10.3390/cells15211934 (PMC13762161; doi:10.3390/cells15211934)
Supplement: Supplementary file 1 [file cells-15-01934-s001.zip › cells-4473515-supplementary.pdf]

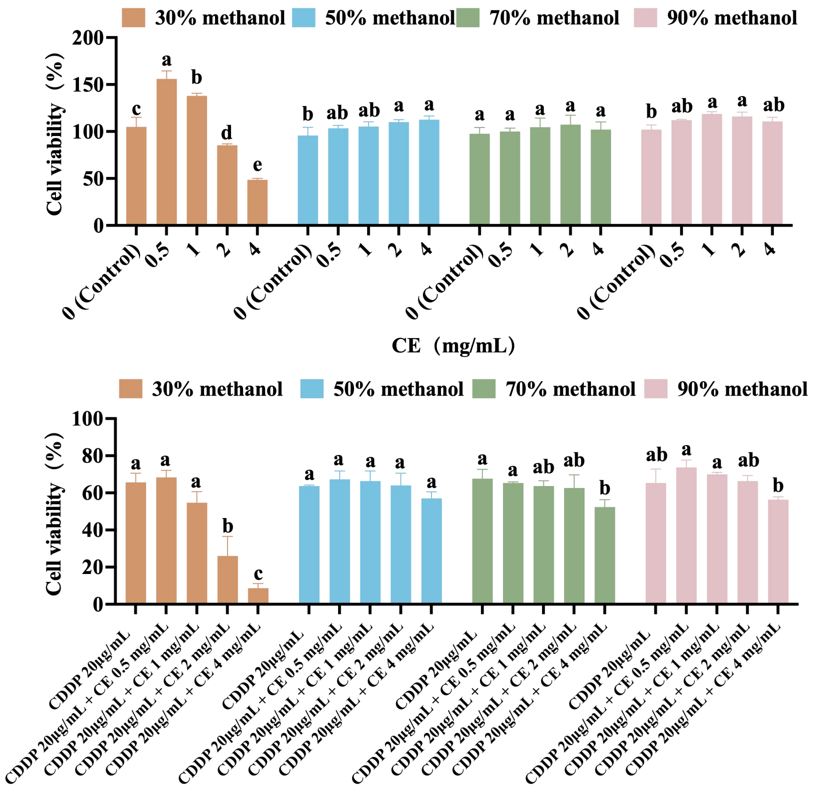

Figure S1. Effects of extraction-solvent concentration on SW620 cell viability. Crude extracts prepared with the solvent concentrations indicated in the plot legends were tested alone (upper panel) or with 20 μg/mL cisplatin (CDDP ; lower panel) for 24 h. Crude extract (CE) concentrations are shown on the x-axes. Data are presented as the mean ± SD ($n = 3$). Bars that do not share a lowercase letter differ significantly ($p < 0.05$).

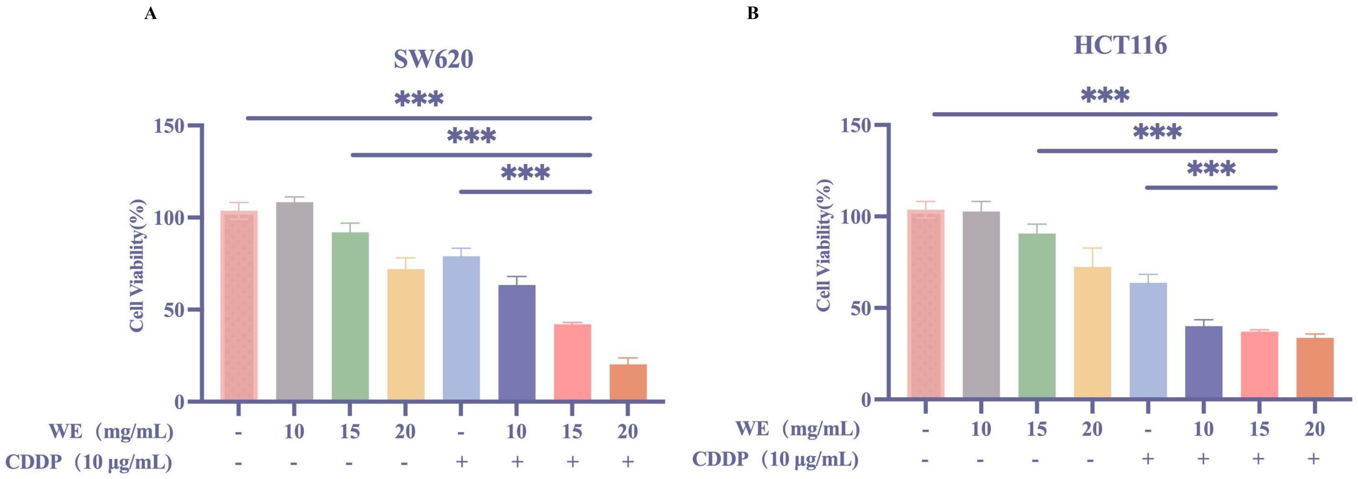

Figure S2. Effects of the residual water extract (WE) and cisplatin (CDDP) , alone or in combination, on colorectal cancer-cell viability after 24 h. (A) SW620 and (B) HCT116 cells were treated with WE (10, 15, or 20 mg/mL), CDDP (10 µg/mL), or the indicated combinations. Data are presented as the mean ± SD (n = 3). Horizontal brackets identify the compared groups; ***$p < 0.001$.

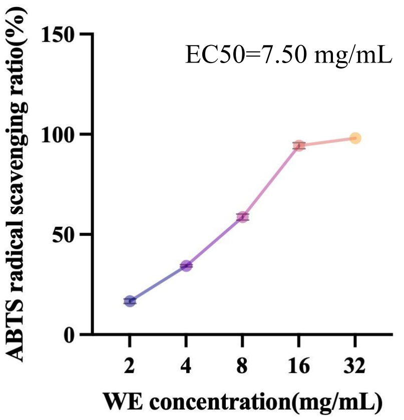

Figure S3. Cell-free 2,2'-azino-bis(3-ethylbenzothiazoline-6-sulfonic acid (ABTS) radical-scavenging activity of the residual water extract (WE) at the indicated concentrations. The estimated EC50 was 7.50 mg/mL. Data are presented as the mean ± SD (*n* = 3).

A

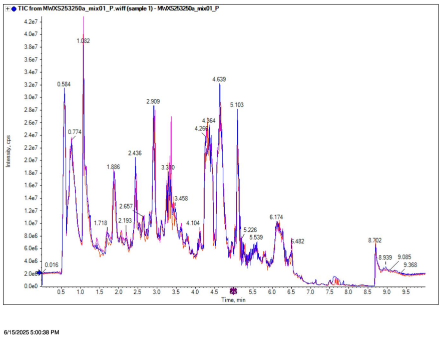

B

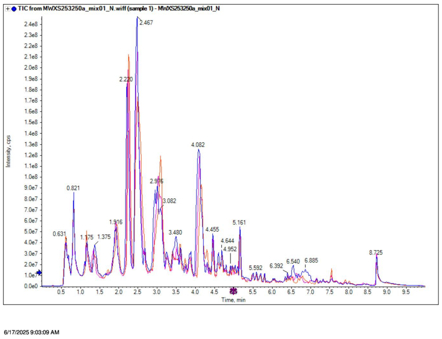

Figure S4. Ultra-performance liquid chromatography-tandem mass spectrometry (UPLC-MS/MS) total-ion chromatograms of the residual water extract (WE) acquired in positive-ion mode (A) and negative-ion mode (B).

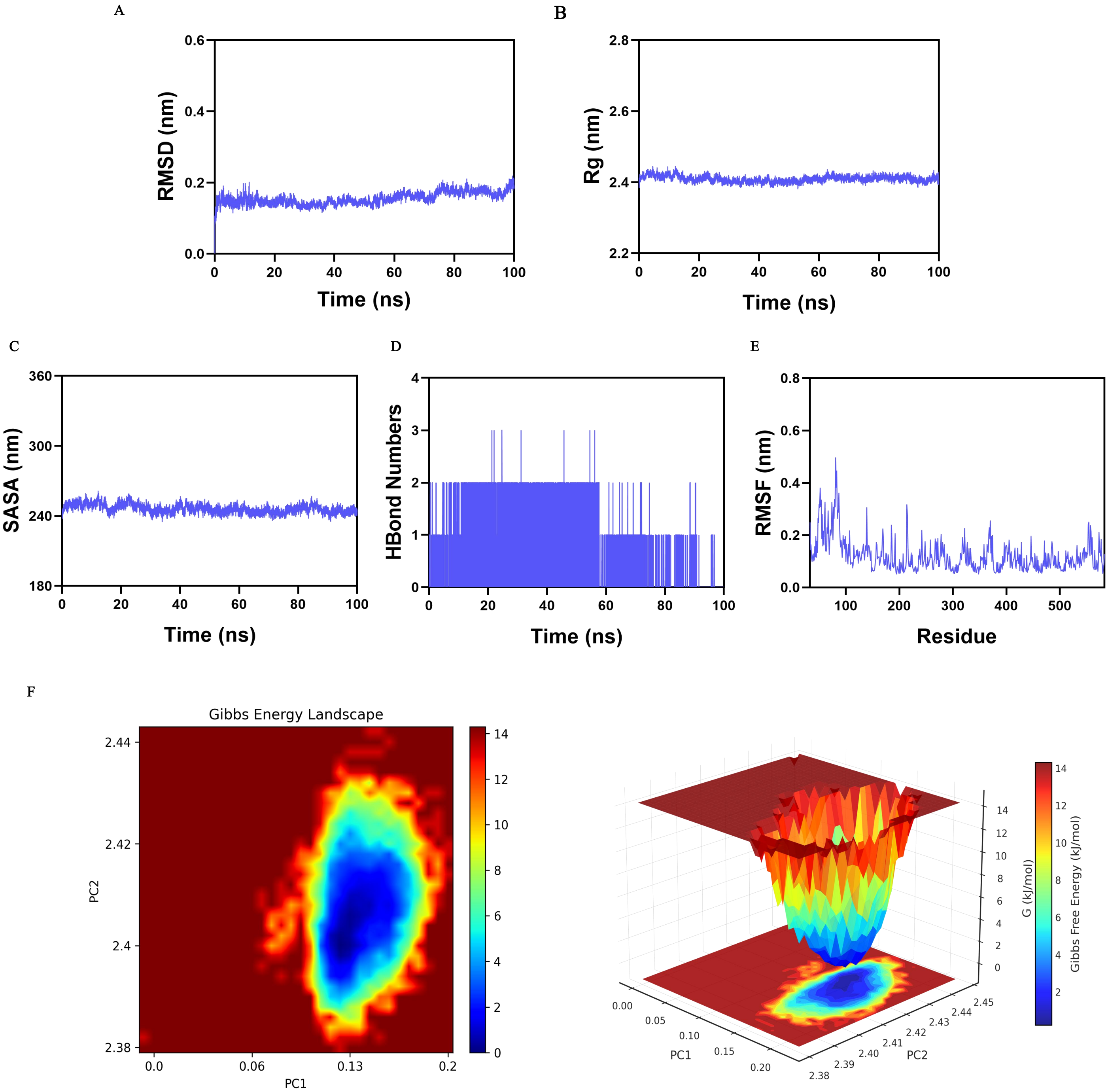

Figure S5. Molecular-dynamics analysis of the docked complex formed by alpha-linolenic acid (ALA) and prostaglandin-endoperoxide synthase 2 (PTGS2) during a 100-ns simulation. (A) Root-mean-square deviation (RMSD). (B) Radius of gyration (Rg). (C) Solvent-accessible surface area (SASA). (D) Number of ligand-protein hydrogen bonds. (E) Root-mean-square fluctuation (RMSF) of PTGS2 residues. (F) Two- and three-dimensional free-energy landscapes based on the first two principal components. These analyses characterize the modeled pose and do not constitute experimental binding validation.

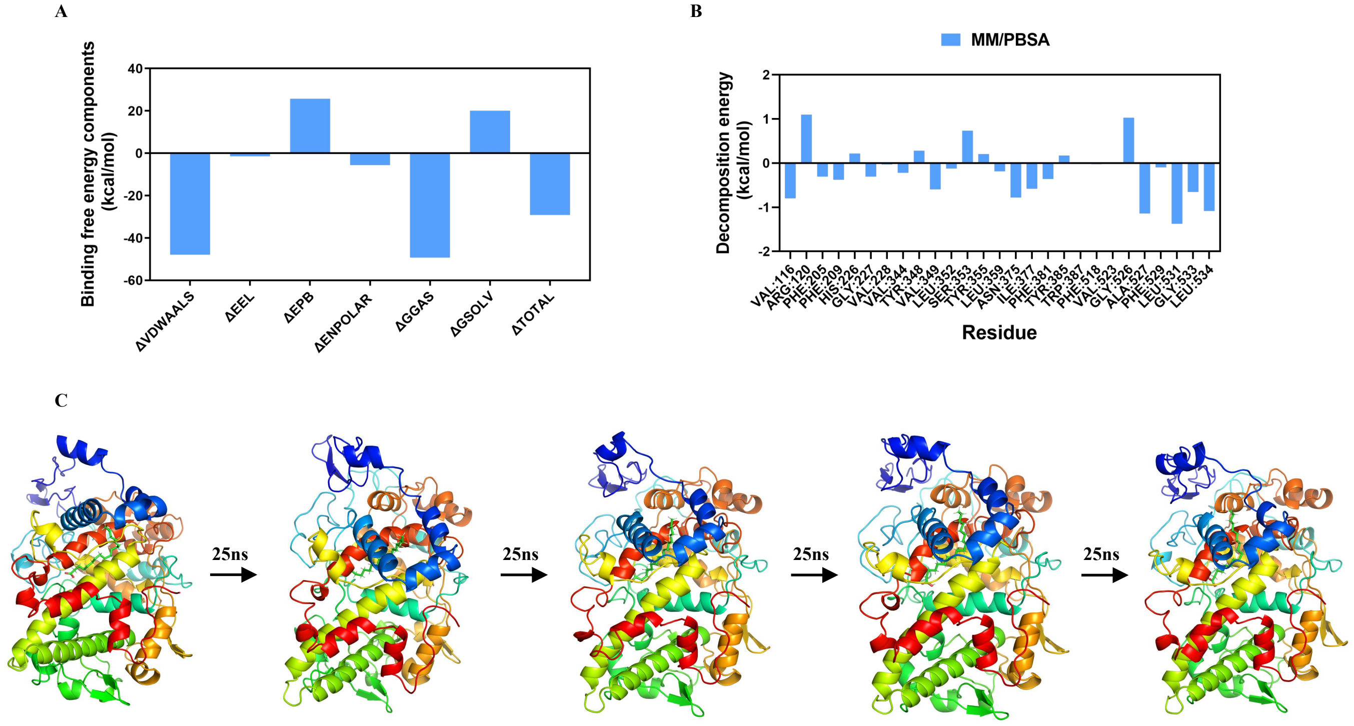

Figure S6. Additional computational analyses of the docked ALA-PTGS2 complex. (A) Molecular mechanics/Poisson-Boltzmann surface area (MM/PBSA) estimates of binding free-energy components. (B) Per-residue decomposition of the calculated interaction energy. (C) Representative structures sampled at 0, 25, 50, 75, and 100 ns during the 100-ns simulation. These results do not establish direct binding or functional modulation in cells.

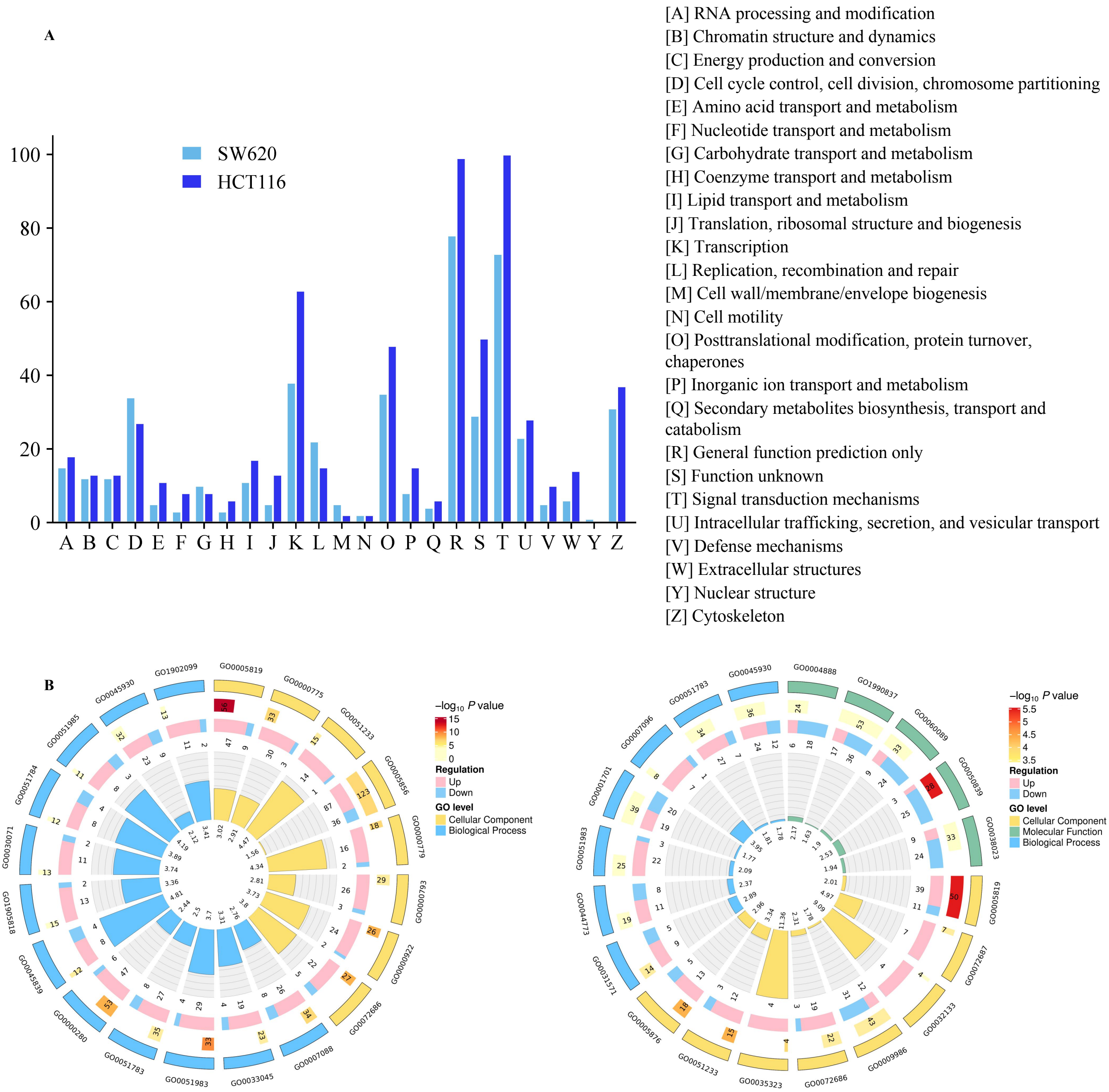

Figure S7. Functional analyses of the quantitative proteomic data from SW620 and HCT116 cells. (A) Clusters of Orthologous Groups (COG) functional classification. (B) Gene Ontology (GO) enrichment analysis of differentially expressed proteins associated with co-treatment using cisplatin (CDDP) and the residual water extract (WE). Enrichment indicates association and does not demonstrate pathway activation.

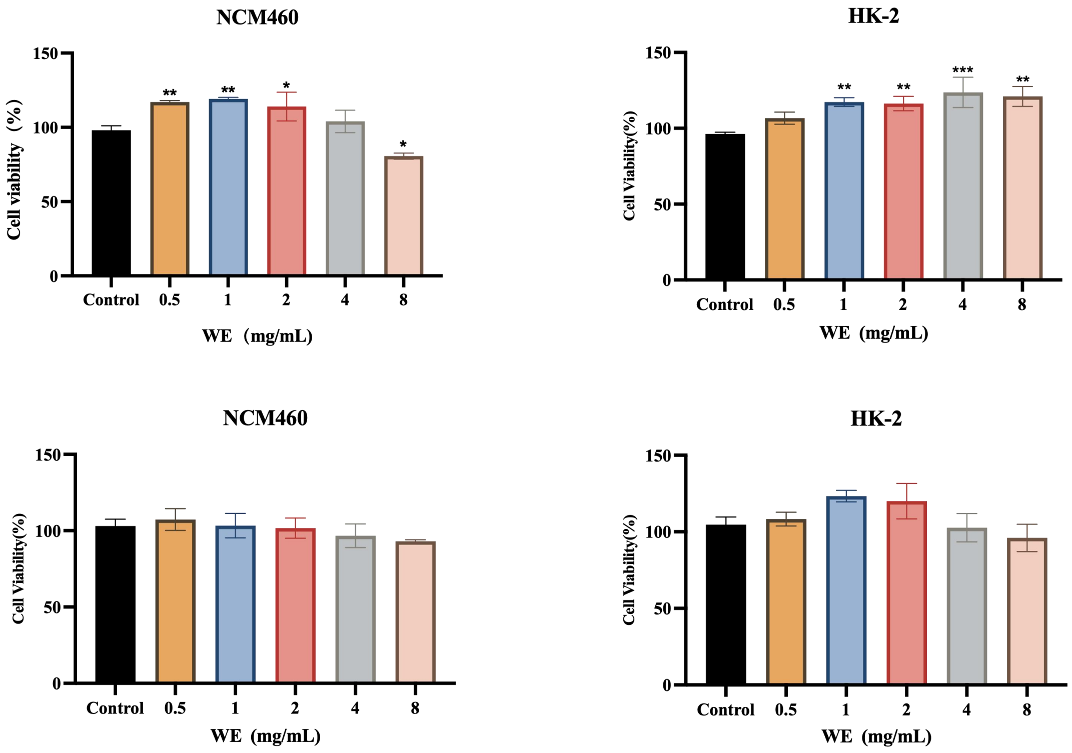

Figure S8. Cell viability of NCM460 and HK-2 cells treated by water extract (WE) with different incubation concentrations for 24 and 48 h.The results were expressed as mean ± SD (n = 3). * $p < 0.05$, ** $p < 0.01$, *** $p < 0.001$ vs. control group.

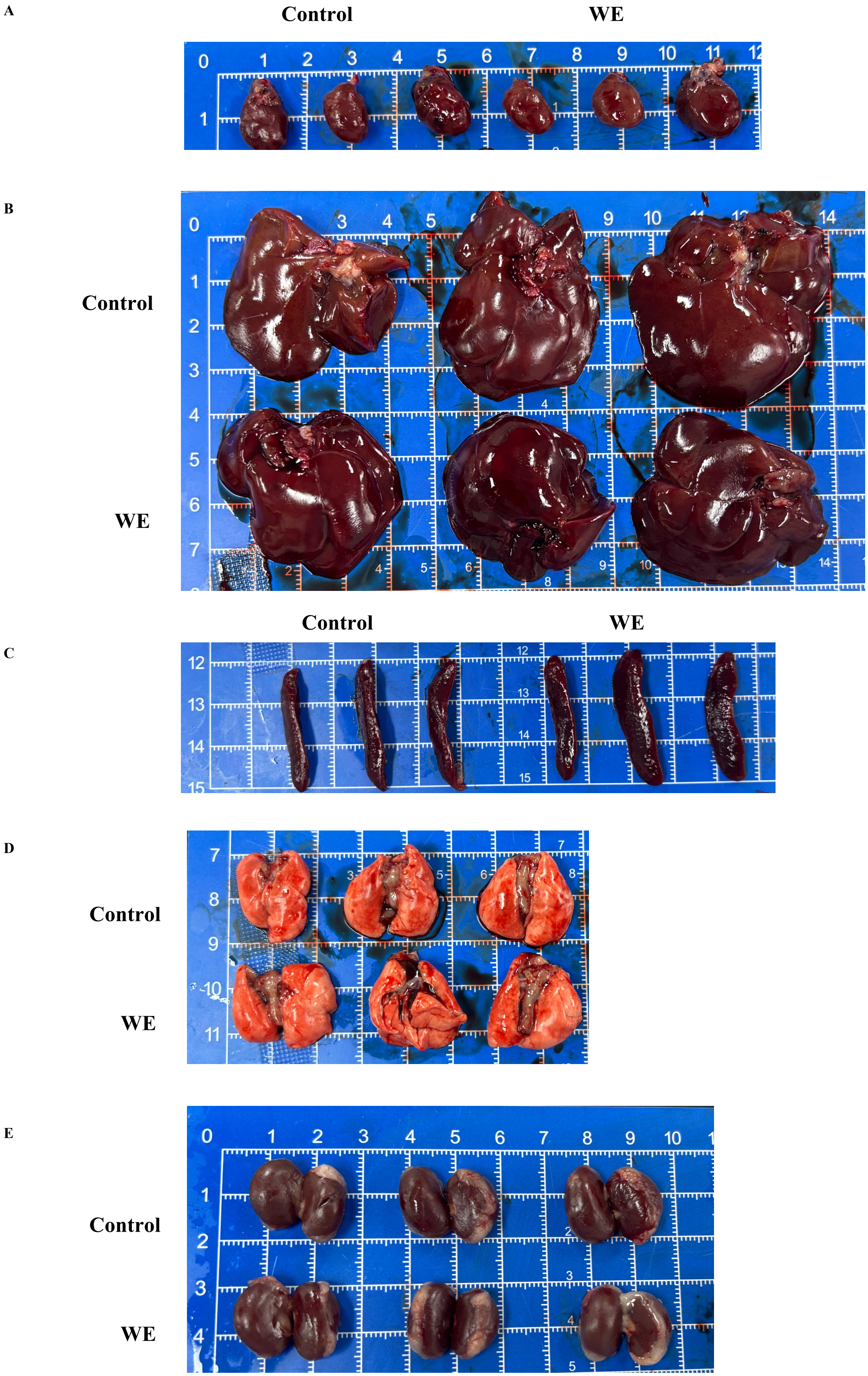

Figure S9. Representative gross appearance of rat major organs after acute toxicity treatment. (A-E) Gross photographs of heart (A), liver (B), spleen (C), lung (D), and kidney (E) collected from rats of control and water extract (WE) groups in acute toxicity assay. Each group contained $n = 3$ rats.

	^{78}Se		$SeCys_2$		MeSeCys		SeIV	SeVI	SeMet
	Content [μg/g]	Extraction rate	Content[μg/g]	Extraction rate	Content[μg/g]	Extraction rate	Content [μg/g]	Content [μg/g]	Content [μg/g]
**SE**	237.54 ± 7.54		29.33 ± 0.18		158.22 ± 0.06		0.44 ± 0.04	0.67 ± 0.04	33.16 ± 0.59
**CE**	471.59 ± 24.91	85.71%	37.99 ± 1.58	55.92%	334.69 ± 12.11	91.32%	-	-	-
**EAE**	341.27 ± 26.32	1.76%	-	-	202.25 ± 12.36	4.03%	-	-	-
**PEE**	642.04 ± 38.16	4.57%	-	-	93.71 ± 3.74	0.27%	-	-	-
**BE**	923.73 ± 21.50	20.83%	102.52 ± 12.73	28.69%	657.26 ± 19.68	20.88%	-	-	-
**WE**	421.5 ± 21.09	64.84%	42.28 ± 1.76	85.94%	321.22 ± 4.9	74.11%	-	-	-
**Total**		92.00%				99.29%			

Table S1. Total selenium content and High-performance liquid chromatography–inductively coupled plasma–mass spectrometry (HPLC-ICP-MS) selenium-speciation results for the selenium-enriched rapeseed starting material (SE), crude extract (CE), ethyl acetate extract (EAE), petroleum ether extract (PEE), n-butanol extract (BE), and water extract (WE).

	MA(%) (C14:0)	PA(%) (C16:0)	SA(%) (C18:0)	OA(%) (C18:1)	LA(%) (C18:2)	ALA(%) (C18:3)	AA(%) (C20:0)	CEA(%) (C20:1)	CA(%) (C22:0)	EA(%) (C22:1)	TA(%) (C24:0)
**SE**	-	11.08 ± 0.34	2.13 ± 0.07	2.67 ± 0.04	13.7 ± 0.03	**69.71 ± 0.33**	-	-	-	0.71 ± 0.06	-

Table S2. Fatty-acid composition of the selenium-enriched rapeseed starting material (SE), expressed as percentages of total measured fatty acids. Data are presented as the mean ± SD.

	WBC (10^9/L)	LYM (10^9/L)	MONO (10^9/L)	NEUT (10^9/L)	EO (10^9/L)	BASO (10^9/L)	RBC (10^{12}/L)	HGB (g/L)	PLT (10^9/L)
**Control**	6.99±2.58	6.9±3.29	0.03±0.01	0.67±0.28	0.03±0.01	0±0	6.56±0.36	151±9.17	1375±134.73
**WE**	6.01±1.33	5.35±1.26	0.05±0.02	0.58±0.08	0.03±0.01	0±0	6.5±0.37	147.33±10.12	1253±160.51

Table S3. Complete blood count analysis results.

Gene	Primer Sequence
ACTB-F	TCACCATGGATGATGATATCGC
ACTB-R	ACATAGGAATCCTTCTGACCC
CASP6-F	GCACCCGGCAGTGTCAA
CASP6-R	ATGACTGGCACATCGTGCTG
CASP9-F	TTTGGTGATGTCGAGCAGAA
CASP9-R	GTGAGCCCACTGCTCAAAGA
JUN-F	GTGCCGAAAAAGGAAGCTGG
JUN-R	GCTGCGTTAGCATGAGTTGG
MAPK14-F	GAAAATCTCCTCAGAGTCTGCAA
MAPK14-R	GATCGGCCACTGGTTCATCA
BAX-F	GCCCTTTTGCTTCAGGGTTTC
BAX-R	GGAAAAAGACCTCTCGGGGG

Table S4. Primer sequences for qRT-PCR.

Binding free energy (kcal/mol)	ALA	I3C	MSC	SFN
BAX	−5.3	−5.2	−3.7	−3.5
BCL2	−6	−5.9	−4	−4
CASP3	−5.1	−5.1	−4.2	−3
CASP9	−5.5	−6.9	−4.3	−4
CDK2	−6.2	−6.2	−3.8	−4
HMOX1	−6.3	−5.7	−3.8	−3.9
JUN	−4.4	−5	−3.3	−3.2
MAPK1	−5	−5.4	−4.1	−3.6
MYC	−5.3	−5.3	−3.6	−2.9
STAT3	−4.7	−4.9	−4.2	−3.2
TP53	−4.8	−6.2	−4.4	−4.1
AKT1	−6.1	−6.3	−4.1	−3.9
CCNB1	−6.2	−6.5	−4.6	−3.5
CCND1	−5.5	−5.8	−3.9	−3.7
EGFR	−6.8	−5.8	−4.1	−4.1
FOS	−4.5	−4.9	−3.5	−3.4
GPX4	−5.4	−5.3	−4.1	−3.9
PTGS2	−7.3	−6.8	−4.5	−4.6
MMP3	−6.9	−6.7	−4.2	−4.1

Table S5. Binding free energy of active ingredients with proteins by molecular docking.
